# Impact of HIV self-testing for oral pre-exposure prophylaxis scale-up on drug resistance and HIV outcomes in western Kenya: a modelling study

**DOI:** 10.1016/S2352-3018(23)00268-0

**Published:** 2024-01-29

**Authors:** Sarah N Cox, Linxuan Wu, Rachel Wittenauer, Samantha Clark, D Allen Roberts, Ifechukwu Benedict Nwogu, Olga Vitruk, Alexandra P Kuo, Cheryl Johnson, Muhammad S Jamil, Anita Sands, Robin Schaefer, Christine Kisia, Rachel Baggaley, Joanne D Stekler, Adam Akullian, Monisha Sharma

**Affiliations:** a Department of Epidemiology, University of Washington, Seattle, WA, USA; b Department of Global Health, University of Washington, Seattle, WA, USA; c Department of Pharmacy, University of Washington, Seattle, WA, USA; d Global HIV, Hepatitis and STIs Programmes, World Health Organization, Geneva, Switzerland; e Regulation and Prequalification Department, World Health Organization, Geneva, Switzerland; f World Health Organization – Kenya Country Office, Nairobi, Kenya; g Department of Medicine, University of Washington, Seattle, WA, USA; h Institute for Disease Modeling, Bill and Melinda Gates Foundation, Seattle, WA, USA

## Abstract

**Background:**

Community-based oral pre-exposure prophylaxis (PrEP) provision has the potential to expand PrEP coverage. HIV self-testing can facilitate PrEP community-based delivery but might have lower sensitivity than facility-based HIV testing, potentially leading to inappropriate PrEP use among people with HIV and subsequent development of drug resistance. We aimed to evaluate the impact of HIV self-testing use for PrEP scale-up.

**Methods:**

We parameterised an agent-based network model, EMOD-HIV, to simulate generic tenofovir disoproxil fumarate and emtricitabine PrEP scale-up in western Kenya using four testing scenarios: provider-administered nucleic acid testing, provider-administered rapid diagnostic tests detecting antibodies, blood-based HIV self-testing, or oral fluid HIV self-testing. Scenarios were compared with a no PrEP counterfactual. Individuals aged 18–49 years with one or more heterosexual partners who screened HIV-negative were eligible for PrEP. We assessed the cost and health impact of rapid PrEP scale-up with high coverage over 20 years, and the budget impact over 5 years, using various HIV testing modalities.

**Findings:**

PrEP coverage of 29% was projected to avert approximately 54% of HIV infections and 17% of HIV-related deaths among adults aged 18–49 years over 20 years; health impacts were similar across HIV testing modalities used to deliver PrEP. The percentage of HIV infections with PrEP-associated nucleoside reverse transcriptase inhibitor (NRTI) drug resistance was 0·6% (95% uncertainty intervals 0·4–0·9) in the blood HIV self-testing scenario and 0·8% (0·6–1·0) in the oral HIV self-testing scenario, compared with 0·3% (0·2–0·3) in the antibody rapid diagnostic testing scenario and 0·2% (0·1–0·2) in the nucleic acid testing scenario. Accounting for background NRTI resistance, we found similarly low proportions of drug resistance across scenarios. The budget impact of implementing PrEP using HIV self-testing and provider-administered rapid diagnostic tests were similar, while nucleic acid testing was approximately 50% more costly.

**Interpretation:**

Scaling up PrEP using HIV self-testing has similar health impacts, costs, and low risk of drug resistance as provider-administered rapid diagnostic tests. Policy makers should consider leveraging HIV self-testing to expand PrEP access among those at HIV risk.

**Funding:**

The Bill and Melinda Gates Foundation.

## Introduction

Despite the high efficacy of oral pre-exposure prophylaxis (PrEP) and availability in clinics,^1^ uptake is below global targets in sub-Saharan Africa.^2^ Barriers to clinic-based PrEP uptake include privacy concerns, lost wages due to long wait and travel times to clinics, stigma, limited clinic hours, and understaffing.^3^ Community-based PrEP provision (eg, via pharmacies, mobile sites, home delivery, or telehealth) is a promising strategy to overcome barriers associated with facility-based PrEP and expand coverage.^3^ Sub-Saharan Africa has a growing network of remote and community-based health services, which expanded during the COVID-19 pandemic. The Kenya Ministry of Health has prioritised the expansion of community HIV prevention services to achieve the UNAIDS target of 10 million people on PrEP by 2025, particularly in regions of high HIV burden, such as western Kenya.

Community-based PrEP delivery requires acceptable HIV testing approaches, as an HIV-negative result is necessary before PrEP initiation and at regular intervals to support PrEP continuation. HIV self-testing can facilitate PrEP delivery outside clinic settings by more easily enabling non-health-care workers to provide HIV testing. HIV self-testing might be more feasibly implemented than provider-administered antibody rapid diagnostic testing in settings such as pharmacies by reducing personnel time and administration costs associated with testing and allowing for increased client privacy by enabling users to conduct their own testing in a private area. Updated guidance from WHO suggests that HIV self-testing can be used for PrEP service delivery,^4^ with six HIV self-testing products currently WHO pre-qualified.^5^ However, HIV self-tests can be associated with user error and might be less sensitive in detecting HIV than provider-administered rapid diagnostic tests.^6^ In field-performance studies of HIV self-tests in sub-Saharan Africa, false HIV-negative test results have accounted for 0·3–1·5% of blood HIV self-tests and 0·5–4·8% of oral HIV self-tests (appendix pp 4–6).^7^, ^8^ Lower test sensitivity can lead to inappropriate PrEP provision to people with HIV (due to a false HIV-negative test result) and subsequent development of nucleoside reverse transcriptase inhibitor (NRTI) drug resistance. The health and economic impact of using HIV self-testing for PrEP scale-up is not well understood, particularly whether HIV self-testing would increase NRTI drug resistance.^1^, ^9^ Additionally, nucleic acid testing has been proposed as an alternative testing approach to deliver PrEP due to its high sensitivity. However, nucleic acid testing is considerably more expensive and generally requires a return visit to obtain results which can lead to loss-to-follow-up.

**Research in context**

**Evidence before this study**

We searched PubMed for modelling studies published from inception to March 29, 2023, that assessed the health or economic impact of pre-exposure prophylaxis (PrEP) scale-up in sub-Saharan Africa using the terms: “HIV” AND “pre-exposure prophylaxis”, “PrEP” AND (a list of terms indicating health impact), “cost*”, “budget impact”, “economic evaluation” AND (a list of countries in sub-Saharan Africa), “sub-Saharan” AND “model*”, OR “mathematical model*”. Our search identified 362 articles. Several studies evaluated the health impact or cost-effectiveness of PrEP scale-up, but few accounted for HIV drug resistance. One compartment model evaluated the effect of PrEP use on drug resistance with general PrEP targeting (ie, not based on sexual partnerships) and recommended frequent HIV testing to limit inappropriate PrEP use and mitigate the spread of HIV drug resistance. Another agent-based modelling study found that making PrEP easily accessible through community-based delivery could be cost-effective in high HIV burden areas if PrEP use was well-aligned with individual's periods of risk.

**Added value of this study**

We evaluated the health and budget impact of PrEP scale-up with HIV testing modalities using an individual-based model. We found that scaling up PrEP can result in a substantial reduction in HIV burden with low risks of PrEP-associated drug resistance; findings were similar across HIV testing modalities.

**Implications of all the available evidence**

Increasing PrEP availability in communities is a promising strategy to expand PrEP use in high HIV burden areas. Scaling-up PrEP using HIV self-testing can result in similar health impacts and cost as provider-administered rapid diagnostic testing. Policy makers could consider leveraging HIV self-testing to expand PrEP access in convenient community settings such as pharmacies.

We aimed to inform policy discussions and WHO guidance on expanded PrEP implementation by modelling widespread community-based PrEP scale-up to people with one or more sexual partners (ie, people at risk of being exposed to HIV through sex) in western Kenya. We assessed scenarios of provider-administered testing and self testing for PrEP implementation in community settings compared with a no PrEP counterfactual scenario to evaluate HIV-related health outcomes, PrEP-associated drug resistance, and budget impact of PrEP provision.

## Methods

### Study design and data sources

We adapted an open-source, stochastic, agent-based microsimulation model developed by the Institute for Disease Modeling.^10^ EMOD-HIV incorporates population demography, HIV disease progression, and heterosexual network-based HIV transmission, configured to match age-specific and sex-specific propensities of forming different types of sexual partnerships. The model simulates HIV transmission and the impact of interventions (eg, antiretroviral therapy and PrEP) on epidemic dynamics. HIV interventions are included in EMOD-HIV through a highly configurable health-care module, including a HIV care cascade with HIV testing, linkage, and retention in care, and a PrEP prevention cascade with uptake, adherence, continuation, and re-engagement. The model tracks health outcomes including HIV infections, HIV-related deaths, and health care use, enabling calculation of HIV-related costs.

The model was calibrated and parameterised with epidemiological data from six counties in western Kenya including fertility, mortality, voluntary male circumcision coverage, and health care-seeking behaviour (appendix pp 6–24). We calibrated the model to empirical data from Kenya on HIV prevalence by age and sex, antiretroviral therapy coverage, population size, and validated to data on HIV incidence. Model calibration was done using an optimisation algorithm that maximises the likelihood of matching observed data. We conducted the modelling with 100 good-fitting model parameter sets. We simulated a mean of 65 486 individuals per model run using monthly time-steps.

### Modelled scenarios

**Figure 1:**
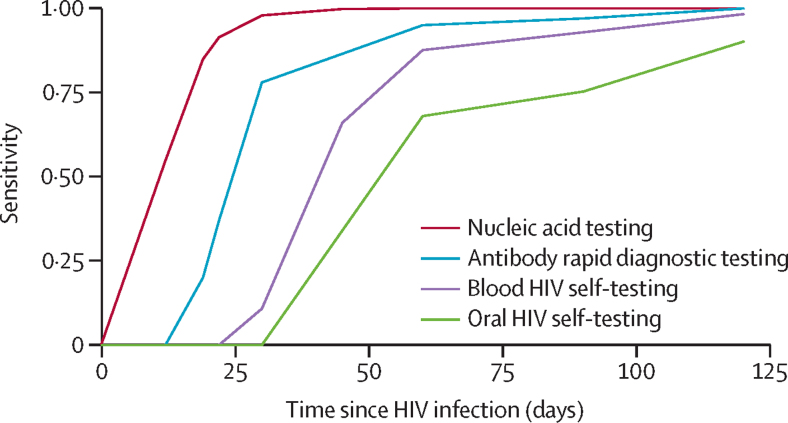
HIV test sensitivity by days since HIV acquisition

In the intervention scenarios, we evaluated community-based PrEP provision (with generic tenofovir disoproxil fumarate and emtricitabine) using four different HIV testing strategies for PrEP initiation and continuation: provider-administered nucleic acid testing; provider-administered antibody rapid diagnostic testing; fingerstick whole blood HIV self-testing; and oral fluid HIV self-testing. We simulated a counterfactual scenario of no PrEP availability. We conducted a literature review and data synthesis in consultation with HIV testing experts to inform assumptions regarding HIV test sensitivity over time since HIV infection, including data from different community-based testing strategies (eg, market centres, village squares, workplace settings, and home).^8^, ^11^, ^12^, ^13^, ^14^ We estimated time-varying HIV test sensitivity for each testing modality and assumed an 11-day delay in HIV detection for individuals on PrEP across all test modalities (figure 1). Details of HIV test sensitivity estimation are provided in the appendix (pp 4–7). Specificity of all HIV testing modalities was assumed to be 100%.

In the PrEP scenarios, we modelled high and rapid scale-up of PrEP among people in partnerships utilising the framework developed by Roberts and colleagues.^15^ Individuals aged 18–49 years entering a heterosexual partnership and not known to have HIV were assumed to have a 75% probability of linking to HIV testing for PrEP initiation; those who tested HIV-negative were assumed to initiate PrEP in the community and those testing HIV-positive were assumed to link to clinic-based confirmatory HIV testing and antiretroviral therapy. People who initiated PrEP had a 75% probability of returning for a PrEP refill after 1 month (and 75% probability of returning for a refill every 3 months thereafter). We used PrEP initiation and continuation probabilities that were higher than those reported in the literature to evaluate the upper bound of impact of PrEP on population-level drug resistance. We assumed individuals underwent HIV testing before PrEP initiation and continuation; HIV testing modality used for PrEP provision varied by scenario. PrEP discontinuation in the model occurred due to loss-to-follow-up or after eligibility criteria were no longer met (ie, an individual turned 50 years old, all their partnerships ended, or they tested HIV-positive). People who discontinued PrEP were eligible to re-initiate upon starting a new partnership if they met eligibility criteria. We assumed PrEP decreased the risk of HIV acquisition by 75%, based on clinical trials accounting for average adherence.^16^

### Outcomes

For each scenario, we assessed the number of HIV infections, HIV-related deaths, PrEP initiations, and people with HIV inappropriately initiated on PrEP over a 20-year time horizon. We estimated PrEP coverage among those aged 18–49 years and person-time on PrEP stratified by HIV status. We also calculated the percentage of HIV infections and HIV-related deaths averted among 18–49-year-olds compared with a scenario of no PrEP use. We designated an individual as inappropriately initiating PrEP if they started PrEP while they had acute (ie, within 1·7 months of HIV infection) or chronic-stage HIV infection. To estimate the HIV-positive person-time on PrEP, we calculated the duration from PrEP initiation for individuals inappropriately initiated on PrEP, or seroconversion for individuals having incident HIV infection while on PrEP, until they discontinued PrEP. We calculated 95% uncertainty intervals (UIs) by computing the 2·5th and 97·5th percentiles across the 100 parameter sets. We conducted a literature review and synthesis of PrEP clinical trials to estimate the probability of developing NRTI drug resistance from PrEP (2·8% among people developing incident HIV infection on PrEP, 32·6% among people initiating PrEP during the acute phase of HIV, and 16·3% among people initiating PrEP with chronic HIV); we applied these probabilities to the number of individuals with HIV on PrEP in each model scenario (appendix pp 42–44).^17^ The prevalence of PrEP-associated NRTI drug resistance was calculated as the total cases of PrEP-associated NRTI resistance divided by the total number of HIV infections. We incorporated background NRTI drug resistance unrelated to PrEP by applying the probability of developing background resistance (30%) to the total number of HIV infections.^18^, ^19^ We calculated the prevalence of population-level NRTI drug resistance by dividing the cases of PrEP-associated and background NRTI drug resistance by the total population. For each simulation, we applied a scaling factor to the modelled population and outcomes to reflect the population size of western Kenya.

### Budget impact analysis

We assessed the undiscounted budget impact of scaling up PrEP by testing modality from the health-care payer perspective over a 5-year time horizon. The main analysis assumes high and rapid scale-up of PrEP, whereas the budget impact analysis uses a lower and more realistic PrEP coverage for budget planning purposes. We assumed individuals aged 18–49 years entering heterosexual partnerships had a 20% probability of initiating PrEP (compared with 75% in the main analysis). We identified financial costs (2021 US$) through literature review supplemented by expert opinion (table 1; appendix pp 25–27), including HIV testing, HIV-related illness, and antiretroviral therapy and PrEP provision. Analysis of model outputs was conducted in R version 4.0.3.

**Table 1:** Model parameters, assumptions, and cost inputs

		**Value**	**Source**
**PrEP**			
PrEP initiation among eligible individuals		75%	Assumption
Efficacy		75%	Baeten et al (2012)^16^
Continuation		75%	Assumption
**Antiretroviral therapy**			
Percentage of people who tested positive through PrEP programmes who initiate antiretroviral therapy		100%	Assumption
Efficacy in reducing HIV transmission		92%	Donnell et al (2017)^20^
**Probability of developing NRTI resistance**			
PrEP-associated resistance			
	Incident HIV infection	2·8%	Gibas et al (2019)^17^
	Acute HIV infection	32·6%	Gibas et al (2019)^17^
	Chronic HIV infection	16·3%	Assumption
Background resistance		30·0%	Gupta et al (2018), Phillips et al (2017)^18^, ^19^
**Costs (2021 US$)**			
**Annual health-care costs (among those not on antiretroviral therapy)**			
	CD4 count <200 cells per μL	$110·30	Eaton et al (2014)^21^
	CD4 count 200–349 cells per μL	$30·38	Eaton et al (2014)^21^
	CD4 count ≥350 cells per μL	$8·59	Eaton et al (2014)^21^
Annual antiretroviral therapy provision costs		$140·89	Phillips et al (2021), Larson et al (2013), The Global Fund (2023)^22^, ^23^, ^24^
End of life care		$105·68	Eaton et al (2014)^21^
PrEP provision (monthly)		$10·66	Kuo et al (2023), Mangale et al (2022)^25^, ^26^
Facility-based HIV-positive test		$3·68	Meisner et al (2021)^27^
Facility-based HIV-negative test		$2·64	Meisner et al (2021)^27^
HIV antibody rapid diagnostic testing		$1·21	Kuo et al (2023), The Global Fund (2023)^25^, ^28^
Blood-based HIV self-testing		$5·00	Expert opinion
Oral fluid HIV self-testing		$3·00	Expert opinion
Nucleic acid test test		$22·00	Expert opinion

PrEP=pre-exposure prophylaxis. Incident infection refers to those who seroconvert while taking PrEP. All HIV test, antiretroviral therapy, and PrEP provision costs include personnel, overheads, and supplies. See additional details on cost parameter calculations in the appendix (pp 25–27).

We evaluated the impact of HIV self-testing in pessimistic and very pessimistic scenarios where we assumed lower sensitivity of HIV tests used for PrEP delivery. In the pessimistic scenarios, we reduced HIV self-testing sensitivities by 30% (ie, multiplied sensitivity by 0·7 across all timepoints). In the very pessimistic scenarios, we used the same test sensitivities as the pessimistic scenarios and further applied a 45-day delay in seroconversion for individuals on PrEP (ie, an additional 34 day delay). We also varied the probability of developing PrEP-associated resistance up to 100%, to evaluate the upper bounds of resistance outcomes.

### Role of the funding source

The funder had no role in the study design, data collection, data analysis, data interpretation, or writing of the report.

## Results

**Figure 2:**
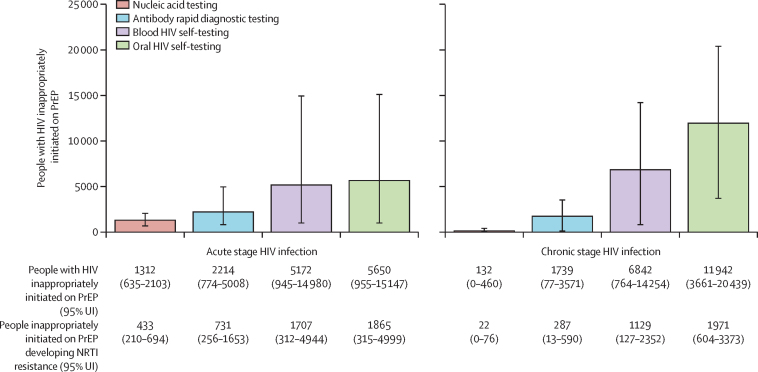
People with HIV inappropriately initiated on PrEP by scenario Assumes a population of 3·7 million individuals. Error bars refer to the 95% uncertainty intervals across the 100 parameter sets. NRTI=nucleoside reverse transcriptase inhibitor. PrEP=pre-exposure prophylaxis.

**Figure 3:**
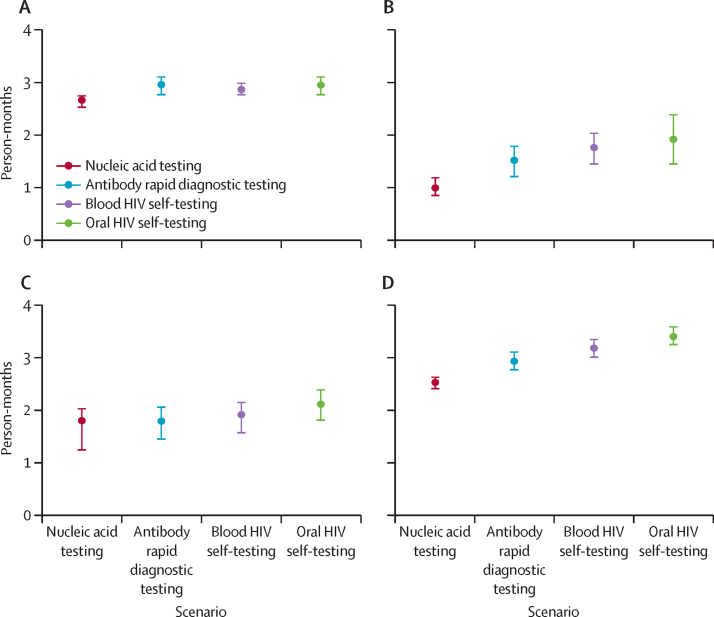
Average person-time inappropriately on PrEP with HIV overall (A), acute stage HIV infection (B), chronic stage HIV infection (C), and incident HIV infection (D) Error bars refer to the 95% uncertainty intervals across the 100 parameter sets. PrEP=pre-exposure prophylaxis.

In all PrEP scenarios, we modelled rapid PrEP scale-up, resulting in an average coverage of 29% among 18–49-year-olds over the 20-year time horizon (appendix p 36). The health impact of community-based PrEP provision was similar across testing scenarios (table 2, figure 2; appendix p 37). Compared with no PrEP, implementing PrEP at high coverage was projected to avert approximately 54% of new HIV infections and 17% of HIV-related deaths over the 20-year time horizon (table 2; appendix p 37). Out of an average scaled population of approximately 3·7 million, the number of people with acute HIV inappropriately initiated on PrEP was 5172 (95% UI 945–14  980) in the blood HIV self-testing scenario and 5650 (955–15 147) in the oral HIV self-testing scenario, compared with 2214 (774–5008) in the antibody rapid diagnostic testing and 1312 (635–2103) in the nucleic acid testing scenarios (figure 2). The number of individuals with chronic HIV inappropriately initiated on PrEP were 6842 (95% UI 764–14 254) in the blood HIV self-testing scenario and 11 942 (3661–20 439) in the oral HIV self-testing scenario, compared with 1739 (77–3571) in the antibody rapid diagnostic testing and 132 (0–460) in the nucleic acid testing scenario. Over 20 years, the total number of people with HIV inappropriately initiating PrEP ranged from 1444 to 17 591 across scenarios (table 2). The average person-time inappropriately taking PrEP while HIV-positive was approximately 3 months, which was similar across HIV self-testing and provider-administered antibody rapid diagnostic testing (figure 3). Nucleic acid testing was associated with slightly shorter person-time on PrEP with HIV both overall (2·67 months) and by acute and incident HIV infection (figure 3).

**Table 2:** Modelled PrEP impact and resistance outcomes by HIV test base case scenario over 20 years among individuals aged 18–49 years

	**No PrEP**	**Self-testing**		**Provider-administered testing**	
		Oral HIV testing	Blood HIV testing	Antibody rapid diagnostic testing	Nucleic acid testing
Infections averted	..	54·2% (49·0–59·6)	54·1% (49·0–59·0)	54·6% (50·2–58·9)	54·7% (50·3–59·7)
Deaths averted	..	17·4% (10·0–24·2)	17·5% (10·4–25·2)	17·9% (8·6–24·1)	17·3% (9·6–24·4)
PrEP initiations (millions)	..	42·8 (40·4–45·2)	42·8 (40·3–45·2)	42·7 (40·3–45·1)	42·8 (40·3–45·0)
Individuals with HIV inappropriately initiated on PrEP (1000s)	..	17·6 (11·0–24·0)	12·0 (7·7–17·3)	4·0 (2·1–5·3)	1·4 (0·7–2·2)
Individuals developing incident HIV while on PrEP (1000s)	..	14·7 (8·1–21·0)	14·7 (8·9–21·3)	14·6 (8·9–20·6)	14·5 (8·0–20·6)
Average person-months on PrEP with undiagnosed HIV	..	3·0 (2·8–3·1)	2·9 (2·8–3·0)	3·0 (2·8–3·1)	2·7 (2·5–2·8)
Individuals with HIV and NRTI resistance (1000s)	185·7 (127·0–237·8)	167·9 (117·4–215·5)	166·9 (117·9–212·3)	164·9 (115·7–210·4)	164·3 (114·7–210·7)
HIV infections with PrEP-associated NRTI resistance	..	0·8% (0·6–1·0)	0·6% (0·4–0·9)	0·3% (0·2–0·3)	0·2% (0·1–0·2)
Population prevalence of NRTI resistance	5·0% (3·4–6·4)	4·5% (3·2–5·8)	4·5% (3·2–5·7)	4·5% (3·1–5·7)	4·4% (3·1–5·7)

Values in parentheses show 95% uncertainty intervals representing the 2·5th and 97·5th percentiles across 100 parameter sets. PrEP=pre-exposure prophylaxis. NRTI=nucleoside reverse transcriptase inhibitor.

Overall, the proportion of HIV infections with PrEP-associated NRTI resistance were 0·6% (95% UI 0·4–0·9) in the blood HIV self-testing scenario and 0·8% (0·6–1·0) in the oral HIV self-testing scenario, compared with 0·3% (0·2–0·3) in the antibody rapid diagnostic testing and 0·2% (0·1–0·2) in the nucleic acid testing scenario (table 2). Accounting for background NRTI resistance (ie, [background resistance cases + PrEP related resistance cases]/population), we found similar prevalence of population-level NRTI resistance across all PrEP scenarios (approximately 4·5%), which was slightly lower than in the no PrEP scenario (5·0%) due to lower HIV incidence in the PrEP scenarios.

HIV infections and deaths averted were similar across pessimistic, very pessimistic (appendix pp 30–35), and base-case scenarios (table 2). Overall, the percentage of HIV infections with PrEP-associated NRTI drug resistance was higher in the pessimistic and very pessimistic scenarios; for example, for oral HIV self-testing, prevalence of drug resistance was 1·1% (pessimistic scenarios) and 1·3% (very pessimistic scenarios), compared with 0·8% in the base-case. Similarly, for blood HIV self-testing, the prevalence of PrEP-associated drug resistance was 1·0% (pessimistic scenarios) and 1·2% (very pessimistic scenarios), compared with 0·6% in the base-case scenario. Accounting for background NRTI drug resistance, the proportion of resistance was similar across scenarios (oral and blood HIV self-testing: 4·5–4·6% *vs* no PrEP: 5·0%). Assuming 100% of people with HIV on PrEP developed drug resistance resulted in a population prevalence of NRTI resistance of less than 6% for all scenarios (appendix pp 32–35).

**Figure 4:**
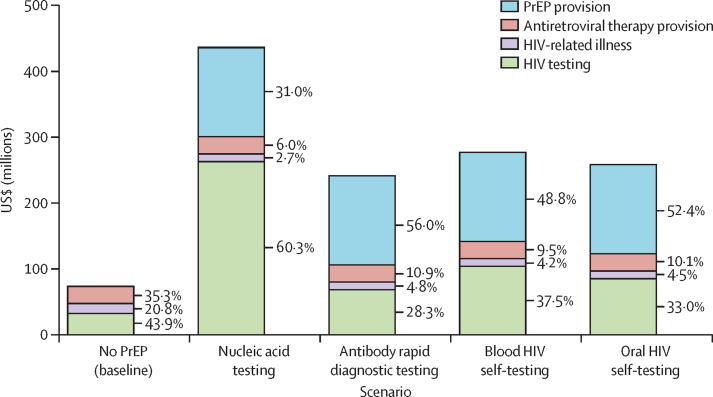
5-year budget impact of PrEP scale-up by HIV testing modality in a population of 3·1 million individuals PrEP=pre-exposure prophylaxis.

Figure 4 shows the 5-year budget impact of PrEP scale-up by testing modality (costs and additional results are in the appendix pp 22–38). In the budget impact analysis scenarios, more realistic modelled PrEP scale-up resulted in a 5·3% PrEP coverage among a population of 3·1 million individuals over the 5-year time horizon (compared with the 29% coverage among 3·7 million individuals in the rapid scale-up PrEP scenarios over 20 years). Costs for PrEP provision were similar for blood HIV self-testing, oral HIV self-testing, and provider-administered antibody rapid diagnostic testing scenarios. PrEP scale-up using nucleic acid testing was approximately 50% more expensive than other testing modalities, mainly due to higher testing costs. The largest component costs of PrEP scale-up were PrEP drugs and provision, with the exception of the nucleic acid testing scenario where HIV testing costs made up the largest component. The component costs were similar in the provider-administered antibody rapid diagnostic testing and HIV self-testing scenarios. Health-care costs for HIV-related illness were lower in the PrEP scenarios (3–5%) compared with the no PrEP scenario (21%).

## Discussion

Our analysis projects that widespread provision of daily oral PrEP in Kenya has the potential to avert more than half of new HIV infections over 20 years, with similar health benefits by HIV testing modality used to deliver PrEP. We modelled high PrEP scale-up and continuation to increase the likelihood of observing more people with HIV inappropriately on PrEP. Despite high PrEP coverage, the population prevalence of NRTI resistance was similar in PrEP scale-up scenarios compared with the counterfactual of no PrEP provision; this is largely due to the reduction in HIV incidence in the PrEP scenarios which results in lower population prevalence of HIV with PrEP-related drug resistance. These findings are consistent with previous modelling studies and suggest that the benefit of utilising HIV self-testing to enable greater PrEP access likely outweighs the risks of PrEP-associated drug resistance.^29^

In addition to drug resistance, another concern about using HIV self-testing for PrEP provision is the potential for people with undiagnosed HIV to continue taking PrEP for long periods before detection, leading to wasted resources (ie, PrEP provision costs) and delays in linkage to antiretroviral therapy. However, we found that despite high continuation rates, people with HIV spent approximately 3 months inappropriately taking PrEP, which was similar for acute, chronic, and incident HIV infections. Findings were robust to changes in assumed delays in time to seroconversion on PrEP and reductions in test sensitivity. Indeed, PrEP scale-up could facilitate linkage to antiretroviral therapy by identifying individuals with undiagnosed HIV through increased HIV testing for PrEP provision.

We explored a theoretical scenario assuming widespread nucleic acid testing for PrEP delivery which has a higher sensitivity, particularly in detecting acute HIV infection. Initiation of PrEP during acute HIV infection is associated with a ten-fold greater risk of NRTI resistance development compared with incident HIV infections while on PrEP.^1^ Although we found a decrease in the number of individuals with HIV inappropriately on PrEP and a slightly shorter person-time inappropriately on PrEP with HIV in the nucleic acid testing scenario, the population-level prevalence of NRTI resistance was similar across HIV testing modalities. This is largely driven by low population prevalence of acute HIV infection in our model, consistent with empirical data. A previous study pooling data across four cohorts in sub-Saharan Africa (composed of female sex workers, men who have sex with men, and people with multiple partners) found a 0·26% prevalence of acute HIV infection;^30^ across six PrEP trials, acute HIV prevalence ranged from 0·1 to 0·4%.^17^ Furthermore, nucleic acid testing is also considerably more costly than other testing modalities, with HIV testing accounting for 60% of PrEP scale-up costs compared with 28–38% for other testing modalities. Additionally, nucleic acid testing is logistically more challenging to implement in community-based settings and usually requires clients to return for a second visit to obtain results before obtaining PrEP, which can result in loss-to-follow-up. Our cost estimates for nucleic acid testing are conservative and do not include scale-up costs, provider training, and assays needed for confirmatory testing. Overall, we find little incremental benefit of implementing nucleic acid testing compared with HIV self-testing or provider-administered rapid diagnostic testing.

Our findings suggest that using blood-based or oral fluid HIV self-testing for PrEP initiation and continuation among the general population has similar health benefits and budget impact compared with provider-administered rapid diagnostic testing. HIV self-testing for community-based PrEP provision is a promising strategy to increase PrEP uptake and retention among those with HIV risk. The SEARCH trial of community health worker-delivered dynamic choice HIV prevention found that offering participants choices for HIV prevention services, including HIV self-testing, substantially increased coverage of biomedical prevention during periods of HIV risk. Half of participants in the intervention group chose HIV self-testing instead of provider-administered rapid diagnostic testing for HIV prevention services, and this proportion increased to 71% by the end of the trial.^31^ A discrete choice experiment assessing user preferences for PrEP delivery via online pharmacies in Kenya found that participants strongly preferred HIV self-testing over provider-administered rapid diagnostic testing.^32^ The increased privacy offered by HIV self-testing might also increase PrEP coverage among key populations, including female sex workers and men who have sex with men.

Although we found little difference in NRTI resistance in the HIV self-testing compared with the rapid diagnostic testing scenarios, the number of people with HIV on PrEP was slightly higher in the HIV self-testing scenarios, with most individuals in the chronic HIV stage. This reflects the relatively larger number of chronically infected individuals in the population compared with those in the acute phase. There is an absence of data from PrEP trials on the probability of resistance development in chronic infection and monitoring of PrEP programmes will be necessary to evaluate inappropriate PrEP use and resistance with large-scale roll-out.

Our findings should be interpreted within the context of several limitations. Our modelled health impacts assume PrEP reaches ambitious levels of scale-up, beyond what would likely be implemented by PrEP programmes. Our main objective was to evaluate the upper bound impact of community-based PrEP scale-up with various testing modalities on PrEP-associated drug resistance. We find little impact on population-level drug resistance with widespread PrEP use. Future analyses are needed with realistic scenarios of PrEP uptake to project the health and economic impact of PrEP provision more accurately. Further research is also needed to understand optimal implementation strategies for community-based PrEP, taking into consideration government regulations and cost recovery for commodities. Additionally, more empirical data are necessary to understand the uptake of PrEP when widely available, such as alignment with periods of risk, adherence, retention, type of user support needed, and variations across sub-populations. Furthermore, we do not explicitly model drug resistance but rather calculate the probability of resistance development outside the model based on the number of individuals inappropriately taking PrEP by infection stage and background levels of resistance; therefore, we do not incorporate the impact of transmitted drug resistance. However, studies suggest that NRTI drug resistance confers a fitness cost relative to wild-type HIV making it less likely to transmit to partners.^9^ With the widespread use of dolutegravir-based antiretroviral therapy regimens, previous studies have shown little impact of PrEP-associated NRTI resistance on antiretroviral therapy effectiveness.^22^ Additionally, there is uncertainty regarding the probability of resistance development given inappropriate PrEP use. However, our results were robust to sensitivity analyses with higher probabilities of resistance development. As PrEP is scaled up and additional data are collected regarding resistance, this analysis should be revisited. Additionally, our model only simulates heterosexual mixing, so we cannot assess the impact of PrEP among men who have sex with men, injection drug users, or other key populations. However, studies demonstrate similar HIV self-testing performance (in terms of conducting and interpreting tests) among key populations and the general population, suggesting HIV self-testing can be a viable strategy for increasing PrEP uptake among key groups.^7^ As with all model-based analyses, our findings are dependent on our model's underlying assumptions which could be subject to bias or misclassification. Furthermore, in the budget impact analysis of nucleic acid testing for PrEP provision, we did not include costs related to expanded laboratory infrastructure, confirmatory assays, and provider training, which would make this scenario more costly. Also, the budget impact analysis utilised costs of community-based PrEP provision at a pharmacy; other strategies such as home or mobile provision might have distinct costs. Finally, although budget impact analysis results project that blood-based HIV self-testing would be slightly more costly than oral fluid HIV self-testing, commodity costs are rapidly changing, as lower cost HIV self-testing kits are approved and there will likely be little difference between blood and oral HIV self-testing. Additional analyses are also needed to explore the impact of leveraging HIV self-testing to reduce the need for quarterly PrEP refill visits. For example, individuals can be prescribed a longer duration of PrEP drugs (6-monthly or 12-monthly) alongside HIV self-tests to use at regular intervals when stopping and restarting PrEP. Future studies are also needed to evaluate the feasibility, acceptability, and demand of community-based PrEP provision for HIV prevention.

Strengths of this analysis include using an individual-based network HIV model and accounting for parameter uncertainty across 100 good-fitting parameter sets. We employed monthly modelled time steps which enabled the identification of individuals initiating PrEP in the acute HIV stage. We targeted PrEP to people with at least one partner to increase the likelihood of PrEP use during periods of HIV risk as has been observed in demonstration projects. We conducted a comprehensive literature review to inform both HIV test sensitivity, which varied by time since infection, as well as the probability of developing NRTI drug resistance, which depended on HIV infection stage. We utilised primary cost data on pharmacy-based PrEP provision with HIV self-testing and provider-administered rapid diagnostic testing from a microcosting in Kenya^25^ to inform the parameters of our budget impact analysis.

Overall, we find that widespread PrEP scale-up using HIV self-testing has the potential to avert substantial HIV burden and has similar costs, health impacts, and NRTI drug resistance as provider-administered rapid diagnostic testing. Our results were robust to a range of sensitivity analyses including a higher risk of resistance development, pessimistic scenarios of HIV self-testing sensitivity, and increased delays in seroconversion on PrEP. As countries strive to reach ambitious targets for PrEP scale-up, community-based strategies which use HIV self-tests can overcome barriers associated with clinic-based PrEP delivery and enable targeted PrEP use during periods of HIV risk in Kenya and similar settings.

## Data sharing

All data used for model calibration are publicly available and can be found in summary tables in the appendix. EMOD-HIV is open-source and available online: https://docs.idmod.org/projects/emod-hiv/en/latest/.

## Declaration of interests

MS reports funding from National Institutes of Health during the conduct of this study. RB reports funds from Unitaid, United States Agency for International Development, and the Bill & Melinda Gates Foundation awarded to WHO. JDS reports receipt of HIV diagnostic device and test kits for conduct of Centers for Disease Control and Prevention-sponsored research from Diagnostics for the Real World. All other authors declare no conflicts of interests. The views expressed in this article do not necessarily represent the views, decisions or policies of the institutions with which they are affiliated.

## References

[1] Fonner VA, Dalglish SL, Kennedy CE (2016). Effectiveness and safety of oral HIV preexposure prophylaxis for all populations. AIDS.

[2] Irungu EM, Baeten JM (2020). PrEP rollout in Africa: status and opportunity. Nat Med.

[3] Ortblad KF, Mogere P, Bukusi E, Ngure K, Baeten JM (2020). Pharmacy delivery to expand the reach of PrEP in Africa. J Int AIDS Soc.

[4] WHO (July 27, 2022). https://www.who.int/publications/i/item/9789240053694.

[5] WHO (2022). https://extranet.who.int/pqweb/vitro-diagnostics/prequalification-reports/whopr?field_whopr_category_tid=60.

[6] Jaspard M, Le Moal G, Saberan-Roncato M, Plainchamp D, Langlois A, Camps P (2014). Finger-stick whole blood HIV-1/-2 home-use tests are more sensitive than oral fluid-based in-home HIV tests. PLoS One.

[7] Majam M, Fischer AE, Rhagnath N (2021). Performance assessment of four HIV self-test devices in South Africa: a cross-sectional study. S Afr J Sci.

[8] Bwana P, Ochieng L, Mwau M (2018). Performance and usability evaluation of the INSTI HIV self-test in Kenya for qualitative detection of antibodies to HIV. PLoS One.

[9] Parikh UM, Mellors JW (2016). Should we fear resistance from tenofovir/emtricitabine preexposure prophylaxis?. Curr Opin HIV AIDS.

[10] Bershteyn A, Gerardin J, Bridenbecker D (2018). Implementation and applications of EMOD, an individual-based multi-disease modeling platform. Pathog Dis.

[11] Kurth AE, Cleland CM, Chhun N (2016). Accuracy and acceptability of oral fluid HIV self-testing in a general adult population in Kenya. AIDS Behav.

[12] Choko AT, Desmond N, Webb EL (2011). The uptake and accuracy of oral kits for HIV self-testing in high HIV prevalence setting: a cross-sectional feasibility study in Blantyre, Malawi. PLoS Med.

[13] Choko AT, MacPherson P, Webb EL (2015). Uptake, accuracy, safety, and linkage into care over two years of promoting annual self-testing for HIV in Blantyre, Malawi: a community-based prospective study. PLoS Med.

[14] Neuman M, Mwinga A, Kapaku K (2022). Sensitivity and specificity of OraQuick® HIV self-test compared to a 4th generation laboratory reference standard algorithm in urban and rural Zambia. BMC Infect Dis.

[15] Roberts DA, Bridenbecker D, Haberer JE, Barnabas RV, Akullian A (2022). The impact of prevention-effective PrEP use on HIV incidence: a mathematical modelling study. J Int AIDS Soc.

[16] Baeten JM, Donnell D, Ndase P (2012). Antiretroviral prophylaxis for HIV prevention in heterosexual men and women. N Engl J Med.

[17] Gibas KM, van den Berg P, Powell VE, Krakower DS (2019). Drug resistance during HIV pre-exposure prophylaxis. Drugs.

[18] Gupta RK, Gregson J, Parkin N (2018). HIV-1 drug resistance before initiation or re-initiation of first-line antiretroviral therapy in low-income and middle-income countries: a systematic review and meta-regression analysis. Lancet Infect Dis.

[19] Phillips AN, Stover J, Cambiano V (2017). Impact of HIV drug resistance on HIV/AIDS-associated mortality, new infections, and antiretroviral therapy program costs in sub-Saharan Africa. J Infect Dis.

[20] Donnell D, Ramos E, Celum C (2017). The effect of oral preexposure prophylaxis on the progression of HIV-1 seroconversion. AIDS.

[21] Eaton JW, Menzies NA, Stover J (2014). Health benefits, costs, and cost-effectiveness of earlier eligibility for adult antiretroviral therapy and expanded treatment coverage: a combined analysis of 12 mathematical models. Lancet Glob Health.

[22] Phillips AN, Cambiano V, Johnson L (2021). Potential impact and cost-effectiveness of condomless-sex-concentrated PrEP in KwaZulu-Natal accounting for drug resistance. J Infect Dis.

[23] Larson BA, Bii M, Henly-Thomas S (2013). ART treatment costs and retention in care in Kenya: a cohort study in three rural outpatient clinics. J Int AIDS Soc.

[24] The Global Fund (October 2023). Pooled procurement mechanism reference pricing: ARVs. https://www.theglobalfund.org/media/5813/ppm_arvreferencepricing_table_en.pdf.

[25] Kuo A, Ekwunife OI, Mogere P (Feb 19–22, 2023). Costs of providing pharmacy-initiated PrEP in Kenya: findings from a pilot study. Conference on Retroviruses and Opportunistic Infections.

[26] Mangale D, Ortblad K, Heitner J (July 29–Aug 2, 2022). Comparing the cost of six-month PrEP dispensing with interim HIV self-testing to the standard-of-care three-month PrEP dispensing with clinic-based testing in Kenya. AIDS.

[27] Meisner J, Roberts DA, Rodriguez P (2021). Optimizing HIV retesting during pregnancy and postpartum in four countries: a cost-effectiveness analysis. J Int AIDS Soc.

[28] The Global Fund (September 2023). Pooled procurement mechanism reference pricing: RDTs. https://www.theglobalfund.org/media/7564/psm_hivrdtreferencepricing_table_en.pdf.

[29] Phillips AN, Bershteyn A, Revill P (2022). Cost-effectiveness of easy-access, risk-informed oral pre-exposure prophylaxis in HIV epidemics in sub-Saharan Africa: a modelling study. Lancet HIV.

[30] Sanders EJ, Wahome E, Powers KA (2015). Targeted screening of at-risk adults for acute HIV-1 infection in sub-Saharan Africa. AIDS.

[31] Kakande E, Ayieko J, Sunday H (Feb 19–22, 2023). Randomized trial of community health worker delivered dynamic choice HIV prevention. Conference on Retroviruses and Opportunistic Infections.

[32] Chen Y, Saldarriaga EM, Montano MA (2023). Assessing preferences for HIV pre-exposure prophylaxis (PrEP) delivery services via online pharmacies in Kenya: protocol for a discrete choice experiment. BMJ Open.

