# Supplementary material for: Impact of HIV self-testing for oral pre-exposure prophylaxis scale-up on drug resistance and HIV outcomes in western Kenya: a modelling study
Source: Lancet HIV. 2024 Jan 29;11(3):e167–75. doi: 10.1016/S2352-3018(23)00268-0 (PMC10896737; doi:10.1016/S2352-3018(23)00268-0)
Supplement: Supplementary appendix [file mmc1.pdf]

THE LANCET
HIV

Supplementary appendix

This appendix formed part of the original submission and has been peer reviewed. We post it as supplied by the authors.

Supplement to: Cox SN, Wu L, Wittenauer R, et al. Impact of HIV self-testing for oral pre-exposure prophylaxis scale-up on drug resistance and HIV outcomes in western Kenya: a modelling study. *Lancet HIV* 2024; published online Jan 29. https://doi.org/10.1016/S2352-3018(23)00268-0.

Online Supplemental Appendix

Accompanying the manuscript:

The impact of HIV self-testing for oral PrEP scale-up on drug resistance and HIV outcomes in western Kenya: a modelling study

Sarah N. Cox, PhD[1,2]*; Linxuan Wu, MHS[1,2]*; Rachel Wittenauer, MPH[2,3]; Samantha Clark, PhD[2,3]; D. Allen Roberts, PhD;[1] Ifechukwu Benedict Nwogu, MPH[2,3]; Olga Vitruk, BS[1,2]; Alexandra P. Kuo, BA[3]; Cheryl Johnson, PhD[4]; Muhammad S. Jamil, PhD[4]; Anita Sands[5]; Robin Schaefer, PhD[4]; Christine Kisia, MPH[6]; Rachel Baggaley, MBBS[4]; Joanne D. Stekler, MD[1,2,7]; Adam Akullian, PhD[2,8]; Monisha Sharma, PhD[2]

Contents

Table S1a. Studies used to develop the adjustment factor for blood-based HIVST field performance

Table S1b. Application of adjustment factor for blood-based HIVST field performance

Table S2a. Studies used to develop the adjustment factor for oral fluid HIVST field performance

Table S2b. Application of adjustment factor for oral fluid HIVST field performance

Table S3. HIV Test Sensitivity Parameter Estimates by Day

Empiric data for model calibration and validation

Table S4a. HIV prevalence in counties of the former Nyanza province (Homa Bay, Kisii, Kisumu, Migori, Nyamira, Siaya), by age and gender[¥]

Table S4b. HIV prevalence among men and women ages 15-49, by county and gender[¥]

Table S5. Number of people on ART by county, gender, and age group[¥]

Table S6. Population size by gender, county, and age group in 2009[¥]

Table S7. Circumcision status quo by county, age group, and year [¥]

Table S8a. Population growth rate among individuals age 15 years+ in the former Nyanza province by year[¥]

Table S8b. Age-specific population fertility rates in Kenya 1950-2049[¥]

Table S8c. Age-specific mortality rates among in Kenya 1950-2049 by gender [¥]

Figure S1. Model fit to age-specific and overall prevalence from population-based surveys by sex

Model Overview and Parameters

Figure S2. EMOD-HIV PrEP cascade

Table S9a. Model parameters. Select model parameters used to fit the EMOD-HIV transmission model to survey data on prevalence and ART coverage from Kenya. Median and interquartile ranges (IQRs) reported for all dynamic parameters used in the calibration process from 100 best-fitting parameter sets. [†]

Table S9b. Cost parameter calculations [¥]

Figure S3. Model predicted population growth by sex

Other Supplemental Tables and Figures

Table S10. Budget impact of 5-year PrEP scale-up by HIV testing modality assuming a population of 3.1 million individuals.

Table S11. Details for modelled PrEP impact and resistance outcomes by HIV test scenario over 20 years among individuals age 18-49 years

Table S12. Sensitivity analysis of modelled PrEP impact and resistance outcomes by test scenario over 20 years among individuals age 18-49 years*

Table S13a. Resistance outcomes at 50% resistance increase and 100% resistance by HIV test scenario over 20 years among individuals age 18-49 years*

Table S13b. Sensitivity analysis of resistance outcomes at 50% resistance increase and 100% resistance by test scenario over 20 years among individuals age 18-49 years*

Figure S4. PrEP coverage over time among 18-49 years old in blood-based HIV self-testing scenario

Figure S5. Proportion of HIV deaths and infection averted by scenario

Figure S6a. Budget impact and cost components of 5-year PrEP scale-up by HIV testing modality, assuming a population of 3.1 million individuals

Figure S6b. Cost components of 5-year PrEP scale-up by HIV testing modality

Figure S7. Modeled HIV incidence by age in western Kenya by scenario

Figure S8. Modeled proportion of acute stage infections among persons not on ART in the population by scenario

Figure S9. Modeled prevalence of population NRTI drug resistance by scenario

Probability of developing PrEP-related HIV drug resistance

HIV Test Sensitivity Parameterization

To estimate the sensitivity of HIV tests over time since HIV infection, we developed a linear spline model including scenarios for HIV self-tests (HIVSTs), rapid diagnostic tests (RDTs), and a nucleic acid test (NAT). The number (n=8) and positions of knots were informed by empiric data available on days since HIV detection (Table S3). For HIVSTs, we included an oral fluid-based self-test and a capillary (fingerstick) blood-based self-test. For RDTs, we included a test that detects antibodies (Ab) to HIV only (3rd generation RDT). For NAT we used the single assay indicated for acute infection.

We utilize nine time points for days since HIV infection in the model (day 0, 12, 19, 22, 30, 45, 60, 90, and 120). We assume a sensitivity of 0 at days 0-12 post-HIV infection for HIVSTs and HIV RDTs during the eclipse phase.(1,2) For individuals on PrEP, we assume an 11-day delay in HIV detection during seroconversion across all test modalities, leading to a shift in test sensitivity. For example, for individuals NOT on PrEP, initial HIV detection with Blood HIVST was possible on day 30 (sensitivity= 0.108); however, among individuals on PrEP this was shifted to an initial HIV detection on day 41 (sensitivity= 0.108).

HIVSTs

To estimate community-based HIVST sensitivity outside of facility settings over time, we applied adjustment factors for field performance to provider-administered HIV test estimates from the Project DETECT seroconversion study(3) and World Health Organization (WHO) prequalification report sensitivity.(4,5) To develop adjustment factors, we identified community-based HIVST sensitivity studies from two systematic reviews (*Pant Pai et al.* 2013 and *Figueroa et al.* 2018).(6,7) These were then supplemented by a literature review of studies published after April 2016. As community-based HIVST sensitivity will depend on the population and implementation strategy, as well as the gold standard, these estimates attempt to account for a range of what has been seen in initial research in settings most similar to Kenya. Only studies from sub-Saharan Africa were included in the development of adjustment factors.

For provider-administered capillary blood-based test sensitivity, we utilized estimates from the *Delaney et al.* 2018 *INSTI HIV-1/HIV-2* fingerstick blood test (bioLytical Laboratories Inc., Canada) at day 30, 45, and 60 post-HIV infection.(3) While this product is only available in the U.S., it represents the closest proxy for sensitivity over time to the rest of world HIVST product, *INSTI HIV Self Test* (bioLytical Laboratories Inc., Canada). For day 120, we used the HIVST sensitivity estimate from the WHO prequalification report for *INSTI HIV Self Test*.(4) For day 90, we used the mean of the sensitivity estimate of day 60 and day 120. For community-based capillary blood HIVST sensitivity, we identified data from two studies using the *INSTI HIV Self Test* conducted in South Africa and Kenya,(8,9) as well one study using *SURE CHECK HIV Self-Test* (Chembio Diagnostic Systems, USA) conducted in South Africa (Table S1a).(8) The adjustment factor for base field performance (0.985) was calculated by taking the mean of these sensitivity estimates divided by the WHO prequalification report sensitivity for *INSTI HIV Self Test* (4)(=0.983/0.998). The adjustment factor was then applied on sensitivity by time from provider-administered blood-based HIV RDT (Table S1b).

Table S1a. Studies used to develop the adjustment factor for blood-based HIVST field performance

Study	Test	Setting	Sensitivity
Majam et al. 2021[8]	*SURE CHECK HIV Self-Test*<br>(Chembio Diagnostic Systems, USA)	South Africa (urban)	0.968
	INSTI HIV Self Test<br>(bioLytical Laboratories Inc., Canada)	South Africa (urban)	0.990
Bwana et al. 2018[9]	*INSTI HIV Self Test*<br>(bioLytical Laboratories Inc., Canada)	Kenya	0.990
			Mean = **0.983**

Table S1b. Application of adjustment factor for blood-based HIVST field performance

Scenario	Sensitivity (days since HIV infection)								
	0	12	19	22	30	45	60	90	120
PrEP with provider-administered Blood HIVST	0	0	0	0 x **0.985**	0.110	0.670	0.890	0.944	0.998
PrEP with self-administered Blood HIVST	0	0	0	0	0.108	0.660	0.876	0.929	0.983

For provider-administered oral fluid-based test sensitivity, we utilized estimates from the *Delaney et al.* 2018 *OraQuick ADVANCE Rapid HIV-1/2 Antibody Test* (OraSure Technologies, USA) oral fluid RDT sensitivity at day 30, 60, and 90 post-HIV infection.(3) This test represented the closest proxy for sensitivity over time to the rest of world HIVST product, *OraQuick HIV Self-Test* (OraSure Technologies, USA).(5) Based on expert opinion, we replaced the reported *OraQuick ADVANCE* day 45 sensitivity of 0.75 with 0.375 in order for the assumed sensitivity of capillary (fingerstick) whole blood RDTs to be higher than oral fluid RDTs.(10) For day 120, we used the HIVST sensitivity estimate of from the WHO prequalification report for *OraQuick HIV Self-Test*.(10) For community-based oral fluid HIVST sensitivity, we identified data from three studies using the *OraQuick HIVST prototype* (OraSure Technologies, USA) conducted in Zambia, South Africa, and Ethiopia,(8,11,12) as well as six studies using *OraQuick ADVANCE Rapid HIV-1/2 Antibody Test* conducted in Kenya, Zimbabwe, South Africa, Malawi, and Uganda (Table S2a).(13-18) We included studies using *OraQuick ADVANCE Rapid HIV-1/2 Antibody Test* because these sensitivity estimates appeared to be more realistic for community settings. The adjustment factor for base field performance (0.907) was calculated by taking the mean of these sensitivity estimates divided by the WHO prequalification report sensitivity for *OraQuick HIV Self-Test*[5] (=0.902/0.994). The adjustment factor was then applied on sensitivity by time from provider-administered oral fluid HIV RDT (Table S2b).

Table S2a. Studies used to develop the adjustment factor for oral fluid HIVST field performance

Study	Test	Setting	Sensitivity
Neuman et al. 2022[11]	*OraQuick HIVST prototype* (OraSure Technologies, USA)	Zambia (rural & urban)	0.875
Majam et al. 2021[8]		South Africa (urban)	0.993
Belete et al. 2019[12]		Ethiopia (urban)	0.995
Kurth et al. 2016[13]	*OraQuick ADVANCE* (OraSure Technologies, USA)	Kenya (urban)	0.897
Mavedzenge et al. 2015[14]		Zimbabwe (rural)	0.667
		Zimbabwe (urban)	0.800
Martínez Pérez et al. 2016[15]		South Africa (rural)	0.988
Choko et al. 2015[16]		Malawi (urban)	0.936
Choko et al. 2011[17]		Malawi (urban)	0.964
Asiimwe et al. 2014[18]		Uganda (rural)	0.900
			Mean = **0.902**

Table S2b. Application of adjustment factor for oral fluid HIVST field performance

Scenario	Sensitivity (days since HIV infection)								
	**0**	**12**	**19**	**22**	**30**	**45**	**60**	**90**	**120**
PrEP with provider-administered Oral HIVST	0	0	0	0	0	0.375	0.750	0.830	0.994
					x 0.907				
PrEP with self-administered Oral HIVST	0	0	0	0	0	0.340	0.680	0.753	0.902

Sensitivity Analyses
To account for the potential worst case performance of HIVSTs, we conducted sensitivity analyses on HIVST sensitivity estimates. In the pessimistic scenarios, we adjust HIVST sensitivities by 70% and keep an 11-day delay in test sensitivity during seroconversion for individuals on PrEP for all test types (i.e., same delay as base scenario).(19-21) In the very pessimistic scenarios, we adjust HIVST sensitivities by 50% and apply a 45-day delay in test sensitivity during seroconversion for individuals on PrEP for all test types (i.e., an additional 34-day delay).

RDTs
For provider-administered Ab RDT sensitivity, we utilized data from *Taylor et al.* 2015(22) on the cumulative probability of a negative test result for a third- & fourth-generation HIV test at various time points in an HIV-positive individual. We first calculated the inverse of cumulative probability to obtain the cumulative probability of a positive test result. To adjust for RDTs (as these were excluded from *Taylor et al.* 2015), we shifted the inverse cumulative probability 10 days forward. Finally, we made an adjustment to Ab RDT at day 45 by using mean of day 30 and 60 (0.865 vs. 0.950).

NAT
To estimate the sensitivity of provider-administered NAT, we utilized results from the simulated eclipse period probability density function from *Delaney et al.* 2017.(23) We extracted *Aptima HIV-1 RNA Qualitative Assay* (Hologic, Inc., USA) NAT detectability by days from HIV acquisition and corresponding density using a data abstraction tool, Plot Digitizer.(24) Additionally, we compared density to reported quantiles and adjusted where necessary to ensure accuracy. We then calculated cumulative density by day to approximate sensitivity over time. In the model we used the same nine input days as other tests.

See Figure 1 (main text) and Table S3 below of sensitivity parameter estimates over time comparing oral fluid HIVST, capillary blood HIVST, Ab RDT, and NAT.

Table S3. HIV Test Sensitivity Parameter Estimates by Day

	Sensitivity (days since HIV infection)								
**Scenario**	**0**	**12**	**19**	**22**	**30**	**45**	**60**	**90**	**120**
No PrEP	0	0	0	0	0	0	0	0	0
PrEP with self-administered Oral HIVST: base	0	0	0	0	0	0.340	0.680	0.753	0.902
PrEP with self-administered Oral HIVST: pessimistic	*0*	*0*	*0*	*0*	*0*	*0.238*	*0.476*	*0.527*	*0.631*
PrEP with self-administered Oral HIVST: very pessimistic	*0*	*0*	*0*	*0*	*0*	*0.170*	*0.340*	*0.376*	*0.451*
PrEP with self-administered Blood HIVST: base	0	0	0	0	0.108	0.660	0.876	0.929	0.983
PrEP with self-administered Blood HIVST: pessimistic	*0*	*0*	*0*	*0*	*0.076*	*0.462*	*0.613*	*0.651*	*0.688*
PrEP with self-administered Blood HIVST: very pessimistic	*0*	*0*	*0*	*0*	*0.054*	*0.330*	*0.438*	*0.465*	*0.491*
PrEP with provider-administered Ab RDT*	0	0	0.200	0.370	0.780	0.865	0.950	0.970	1
PrEP with provider-administered NAT*	0	0.548	0.849	0.914	0.979	0.998	1	1	1

* Theoretical upper bound scenarios to assess the increase in NRTI resistance associated with HIVST

Empiric data for model calibration and validation

Table S4a. HIV prevalence in counties of the former Nyanza province (Homa Bay, Kisii, Kisumu, Migori, Nyamira, Siaya), by age and gender[¥]

Age group	2003		2007		2008		2012	
	Men	Women	Men	Women	Men	Women	Men	Women
**15 - 19**	0.0015	0.0459	0.0121	0.0773	0.0184	0.1078	0.0151	0.0486
**20 - 24**	0.0562	0.2997	0.0257	0.2056	0.0578	0.1201	0.0289	0.1411
**25 - 29**	0.2429	0.2301	0.1956	0.2454	0.2450	0.2228	0.2107	0.2454
**30 - 34**	0.1840	0.1632	0.2578	0.2576	0.1530	0.2593	0.2392	0.2047
**35- 39**	0.2064	0.1838	0.2384	0.2227	0.2275	0.2259	0.1955	0.2811
**40 - 44**	0.2533	0.3500	0.2024	0.1799	0.2501	0.0927	0.3132	0.1694
**45 - 49**	0.1624	0.1651	0.2103	0.1291	0.1331	0.1716	0.1623	0.2287
**15 - 49**	0.1160	0.1830	0.1140	0.1760	0.1140	0.1600	0.1340	0.1760

[¥]**Sources:** Kenya Demographic and Health Surveys, 2003 & 2008; Kenya AIDS Indicator Surveys, 2007 & 2012

Table S4b. HIV prevalence among men and women ages 15-49, by county and gender[¥]

County	2003		2007		2008		2012	
	Men	Women	Men	Women	Men	Women	Men	Women
**Homa Bay**	0.1097	0.2458	0.2514	0.3259	0.1737	0.2524	0.2217	0.2787
**Kisii**	0.0114	0.0853	0.0445	0.0693	0.0330	0.0573	0.0346	0.0368
**Kisumu**	0.1663	0.1914	0.1139	0.1847	0.1109	0.1810	0.1940	0.2022
**Migori**	0.1804	0.1860	0.1685	0.2181	0.1923	0.2228	0.1435	0.1925
**Nyamira**	0.0029	0.0742	-	-	0.0234	0.0544	0.0419	0.1045
**Siaya**	0.1824	0.2424	0.1445	0.2130	0.1526	0.1921	0.2596	0.2990

[¥]**Sources:** Kenya Demographic and Health Surveys, 2003 & 2008; Kenya AIDS Indicator Surveys, 2007 & 2012

Table S5. Number of people on ART by county, gender, and age group[¥]

Gender	County	Age group	Year 2004	2005	2006	2007	2008	2009	2010	2011	2012	2013	2014	2015	2016	2017
Men	Homa Bay	0 - 14	-	-	-	-	-	-	-	-	-	-	2,945	3,583	4,109	4,192
		15 - 99	1,067	2,313	5,148	7,194	10,002	14,436	17,178	15,954	17,522	18,279	19,157	22,834	26,441	29,220
	Kisii	0 - 14	-	-	-	-	-	-	-	-	-	-	828	993	1,109	1,083
		15 - 99	-	-	-	-	-	-	-	2,972	-	-	4,614	5,451	6,604	7,169
	Kisumu	0 - 14	-	-	-	-	-	-	-	-	-	-	3,101	3,245	3,525	3,607
		15 - 99	945	2,047	4,557	6,368	8,853	12,779	15,206	14,122	15,511	16,180	21,216	24,550	28,082	31,021
	Migori	0 - 14											2,309	2,295	2,678	2,673
		15 - 99	711	1,541	3,430	4,793	6,664	9,619	11,446	10,630	11,675	12,179	13,929	15,165	17,438	18,455
	Nyamira	0 - 14	-	-	-	-	-	-	-	-	-	-	484	552	578	611
		15 - 99	-	-	-	-	-	-	-	1,362	-	-	2,120	2,585	3,142	3,474
	Siaya	0 - 14	-	-	-	-	-	-	-	-	-	-	2,645	2,950	3,017	3,197
		15 - 99	860	1,864	4,148	5,797	8,060	11,633	13,843	12,856	14,120	14,730	16,163	18,611	21,477	23,762
Women	Homa Bay	0 - 14	-	-	-	-	-	-	-	-	-	-	3,431	3,835	4,426	4,535
		15 - 99	1,359	2,944	6,551	9,155	12,522	17,202	21,798	31,454	35,182	38,819	40,118	49,956	57,286	61,811
	Kisii	0 - 14	-	-	-	-	-	-	-	-	-	-	906	1,079	1,200	1,146
		15 - 99	-	-	-	-	-	-	-	7,902	-	-	11,691	14,350	17,274	19,044
	Kisumu	0 - 14	-	-	-	-	-	-	-	-	-	-	3,241	3,393	3,810	3,831
		15 - 99	1,203	2,606	5,799	8,104	11,084	15,227	19,296	27,843	31,143	34,362	41,230	48,424	56,384	60,789
	Migori	0 - 14	-	-	-	-	-	-	-	-	-	-	2,526	2,448	2,868	2,884
		15 - 99	905	1,961	4,365	6,100	8,343	11,461	14,524	20,958	23,442	25,865	27,896	31,964	38,637	40,891
	Nyamira	0 - 14	-	-	-	-	-	-	-	-	-	-	506	567	601	622
		15 - 99	-	-	-	-	-	-	-	3,766	-	-	5,964	7,210	8,258	8,654
	Siaya	0 - 14	-	-	-	-	-	-	-	-	-	-	2,778	3,136	3,299	3,569
		15 - 99	1,095	2,372	5,279	7,377	10,090	13,862	17,566	25,347	28,351	31,281	33,911	39,853	44,892	48,808
**Both**	**All**	15-99	8,954	19,404	43,185	60,350	83,142	116,787	143,877	175,003	194,552	210,769	246,293	301,029	333,333	389,159

[¥]Source: Kenya Ministry of Health

Table S6. Population size by gender, county, and age group in 2009[¥]

Age Group	Men						Women					
	Homa Bay	Kisii	Kisumu	Migori	Nyamira	Siaya	Homa Bay	Kisii	Kisumu	Migori	Nyamira	Siaya
**0 - < 1**	18,335	18,236	17,457	19,265	10,313	15,093	18,354	17,993	16,926	19,309	10,263	14,860
**1 - 4**	69,799	69,529	63,054	69,921	41,165	56,269	69,250	69,023	63,172	69,519	40,396	55,901
**5 - 9**	75,926	76,757	67,083	73,872	46,450	60,966	75,973	75,778	67,779	74,333	46,867	60,710
**10 - 14**	68,689	68,473	62,706	64,300	42,590	58,296	67,159	68,072	63,359	63,249	42,198	56,248
**15 - 19**	57,430	59,228	55,597	53,075	36,604	49,220	54,119	60,776	56,741	52,238	36,786	47,825
**20 - 24**	39,573	41,898	47,281	38,690	24,409	32,725	50,309	58,225	57,649	48,004	34,184	41,443
**25 - 29**	30,437	32,792	40,964	30,727	19,515	25,961	36,016	42,878	40,614	34,670	27,273	30,135
**30 - 34**	23,259	26,678	30,412	23,344	16,605	20,359	26,342	30,031	27,515	25,630	19,487	22,328
**34 - 39**	16,013	21,766	21,251	17,024	14,039	14,793	20,010	26,051	20,611	19,313	17,106	17,932
**40 - 44**	11,914	15,718	15,145	12,170	10,470	11,118	16,513	18,360	16,894	14,773	11,377	16,082
**45 - 49**	11,124	16,797	13,361	10,549	11,318	10,390	15,248	19,181	15,298	12,888	11,886	15,486
**50 - 54**	9,705	12,789	11,251	8,565	8,379	9,079	12,942	14,136	12,504	10,314	8,703	14,541
**55 - 59**	8,159	9,527	8,718	6,399	5,999	8,414	9,833	9,528	9,175	7,692	5,819	12,265
**60 - 64**	6,989	7,395	7,054	5,250	5,026	7,712	8,587	7,654	7,597	6,000	5,107	11,081
**65 - 69**	4,325	4,637	4,163	3,382	3,094	5,107	5,957	5,320	5,402	4,508	3,322	7,732
**70 - 74**	4,029	3,945	3,777	2,907	2,753	5,175	5,355	5,017	4,757	3,524	3,153	7,173
**75 - 79**	2,835	2,743	2,392	2,033	1,778	3,549	3,891	3,338	3,356	2,968	1,919	5,464
**80 - 99**	3,726	3,701	2,821	2,624	2,393	4,159	5,316	5,891	4,615	3,636	3,475	6,155

[¥]Source: Kenya National Bureau of Statistics, 2009 Census

Table S7. Circumcision status quo by county, age group, and year [¥]

	Homa Bay			Kisii			Kisumu			Migori			Nyamira			Siaya		
**Year**	**10-14**	**15-24**	**25-49**	**10-14**	**15-24**	**25-49**	**10-14**	**15-24**	**25-49**	**10-14**	**15-24**	**25-49**	**10-14**	**15-24**	**25-49**	**10-14**	**15-24**	**25-49**
**Pre-2008**	0.249	0.249	0.249	0.948	0.948	0.948	0.322	0.322	0.322	0.410	0.410	0.410	0.965	0.965	0.965	0.252	0.252	0.252
**2008**	0.118	0.235	0.275	0.948	0.948	0.948	0.152	0.303	0.333	0.194	0.385	0.451	0.965	0.965	0.965	0.119	0.243	0.270
**2009**	0.122	0.240	0.283	0.948	0.948	0.948	0.178	0.315	0.339	0.199	0.388	0.463	0.965	0.965	0.965	0.127	0.250	0.277
**2010**	0.166	0.285	0.299	0.948	0.948	0.948	0.324	0.389	0.362	0.214	0.395	0.478	0.965	0.965	0.965	0.222	0.310	0.293
**2011**	0.226	0.355	0.313	0.948	0.948	0.948	0.450	0.479	0.385	0.280	0.423	0.486	0.965	0.965	0.965	0.293	0.375	0.304
**2012**	0.337	0.486	0.339	0.948	0.948	0.948	0.516	0.559	0.409	0.516	0.530	0.509	0.965	0.965	0.965	0.417	0.483	0.324
**2013**	0.368	0.565	0.361	0.948	0.948	0.948	0.564	0.634	0.436	0.587	0.614	0.528	0.965	0.965	0.965	0.435	0.549	0.340
**2014**	0.471	0.710	0.399	0.948	0.948	0.948	0.620	0.712	0.467	0.717	0.726	0.554	0.965	0.965	0.965	0.537	0.659	0.368
**2015**	0.506	0.811	0.438	0.948	0.948	0.948	0.672	0.790	0.502	0.742	0.813	0.580	0.965	0.965	0.965	0.562	0.739	0.395
**2016**	0.476	0.894	0.488	0.948	0.948	0.948	0.756	0.844	0.538	0.697	0.891	0.613	0.965	0.965	0.965	0.628	0.841	0.425
**2017**	0.489	0.937	0.537	0.948	0.948	0.948	0.863	0.889	0.572	0.680	0.949	0.648	0.965	0.965	0.965	0.759	0.917	0.457
**2018 onwards**	0.489	0.937	0.537	0.948	0.948	0.948	0.863	0.889	0.572	0.680	0.949	0.648	0.965	0.965	0.965	0.759	0.917	0.457

[¥]**Source:** Circumcision prevalence prior to 2008 is obtained from the Kenya Demographic and Health Survey, 2003. Prevalence of circumcision from 2008 onward combines prevalence of traditional male circumcision and voluntary medical male circumcision estimates obtained from the Decision-Makers' Program Planning Toolkit 2.

Table S8a. Population growth rate among individuals age 15 years+ in the former Nyanza province by year[¥]

Year	Men	Women	Overall growth rate
2019	1,691,466	1,949,646	1.028
2020	1,738,009	2,003,294	1.028
2021	1,785,985	2,058,592	1.028
2022	1,833,807	2,113,713	1.027
2023	1,882,140	2,169,424	1.026
2024	1,932,468	2,227,434	1.027
2025	1,985,507	2,288,569	1.027
2026	2,038,955	2,350,175	1.027
2027	2,097,007	2,417,087	1.028
2028	2,158,280	2,487,713	1.029
2029	2,220,577	2,559,518	1.029
2030	2,281,850	2,630,144	1.028
2031	2,342,510	2,700,063	1.027
2032	2,402,147	2,768,803	1.025
2033	2,460,658	2,836,245	1.024
2034	2,518,607	2,903,039	1.024
2035	2,575,994	2,969,185	1.023
2036	2,631,948	3,033,680	1.022
2037	2,687,288	3,097,468	1.021
2038	2,742,271	3,160,842	1.020
2039	2,797,151	3,224,099	1.020
2040	2,852,185	3,287,533	1.020
2041	2,907,014	3,350,731	1.019
2042	2,962,047	3,414,165	1.019

[¥]**Source**: 2019 population for former Nyanza province based on the Kenya Population and Housing Census. Annual growth rates based on World Population Prospects, assuming growth in the former Nyanza province is similar to national growth rate.

Table S8b. Age-specific population fertility rates in Kenya 1950-2049[¥]

	Age-specific fertility rates (births per 1,000 women)						
**Year**	**15-19**	**20-24**	**25-29**	**30-34**	**35-39**	**40-44**	**45-49**
**1950-1955**	169.1	351.6	338.1	284.3	203.5	110.7	38.9
**1955-1960**	175.9	365.9	351.9	295.8	211.8	115.2	40.5
**1960-1965**	182.3	379.1	364.5	306.5	219.4	119.4	41.9
**1965-1970**	183.3	381.2	366.6	308.2	220.6	120	42.2
**1970-1975**	180.6	375.5	361.1	303.6	217.3	118.3	41.5
**1975-1980**	172.7	359.1	345.3	290.3	207.8	113.1	39.7
**1980-1985**	163.1	339.2	326.2	274.2	196.3	106.8	37.5
**1985-1990**	147.8	307.3	295.5	248.4	177.8	96.8	34.0
**1990-1995**	115.3	268.9	252.0	206.8	161.6	73.4	52.0
**1995-2000**	111.5	260.7	253.3	196.2	143.3	62.4	42.7
**2000-2005**	104.2	243.6	236.7	183.4	133.9	58.3	39.9
**2005-2010**	97.1	227.1	221.4	170.6	123.7	53.6	36.5
**2010-2015**	86.2	201.9	202.3	149.2	102.1	42.4	27.9
**2015-2020**	75.1	176.5	179.8	129.6	85.8	34.8	22.4
**2020-2024**	69.9	165.1	171.8	120.2	76.2	29.9	18.6
**2025-2029**	65.0	154.7	164.8	112.6	68.5	26.0	15.5
**2030-2034**	60.6	145.7	159.1	106.7	62.5	22.9	13.1
**2035-2039**	56.2	137.2	153.6	101.8	57.6	20.4	11.0
**2040-2044**	52.2	129.7	149.3	98.2	53.8	18.5	9.4

[¥]**Source**: 2019 World Population Prospects

Table S8c. Age-specific mortality rates among in Kenya 1950-2049 by gender ¥

Gender	Year	Age-specific mortality rates (%)						
		**15-19**	**20-24**	**25-29**	**30-34**	**35-39**	**40-44**	**45-49**
**Men**	**2000-2004**	0.403	0.624	0.864	1.162	1.596	1.929	2.259
	**2005-2009**	0.293	0.445	0.543	0.676	0.872	1.079	1.336
	**2010-2014**	0.2	0.308	0.393	0.51	0.684	0.861	1.094
	**2015-2019**	0.149	0.231	0.301	0.401	0.552	0.703	0.916
	**2020-2024**	0.131	0.204	0.268	0.36	0.502	0.65	0.858
	**2025-2029**	0.119	0.186	0.243	0.327	0.458	0.6	0.801
	**2030-2034**	0.11	0.172	0.223	0.3	0.422	0.558	0.752
	**2035-2039**	0.102	0.161	0.207	0.279	0.392	0.521	0.708
	**2040-2044**	0.096	0.152	0.195	0.261	0.366	0.489	0.668
**Women**	**2000-2004**	0.266	0.490	0.890	1.232	1.614	1.668	1.760
	**2005-2009**	0.201	0.333	0.519	0.676	0.864	0.980	1.101
	**2010-2014**	0.141	0.235	0.353	0.453	0.583	0.699	0.821
	**2015-2019**	0.102	0.174	0.258	0.330	0.428	0.532	0.645
	**2020-2024**	0.086	0.148	0.222	0.288	0.380	0.481	0.594
	**2025-2029**	0.074	0.128	0.192	0.251	0.336	0.433	0.544
	**2030-2034**	0.065	0.112	0.168	0.221	0.299	0.393	0.501
	**2035-2039**	0.058	0.099	0.149	0.196	0.267	0.357	0.461
	**2040-2044**	0.052	0.089	0.134	0.176	0.241	0.326	0.425

¥**Source**: 2019 World Population Prospects

Figure S1. Model fit to age-specific and overall prevalence from population-based surveys by sex

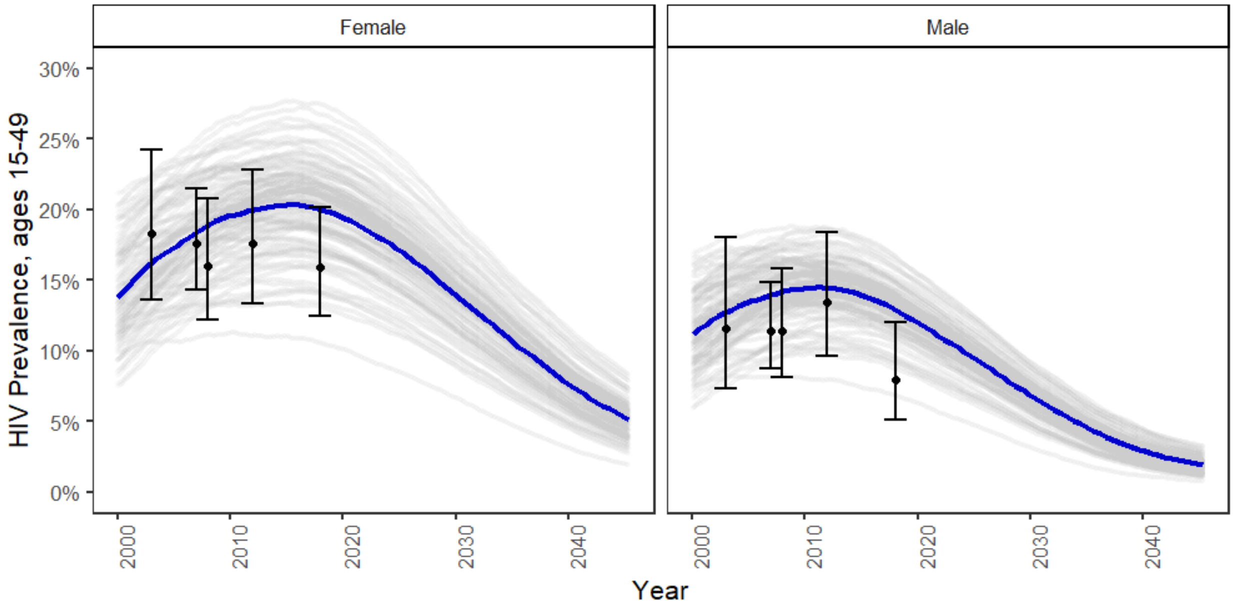

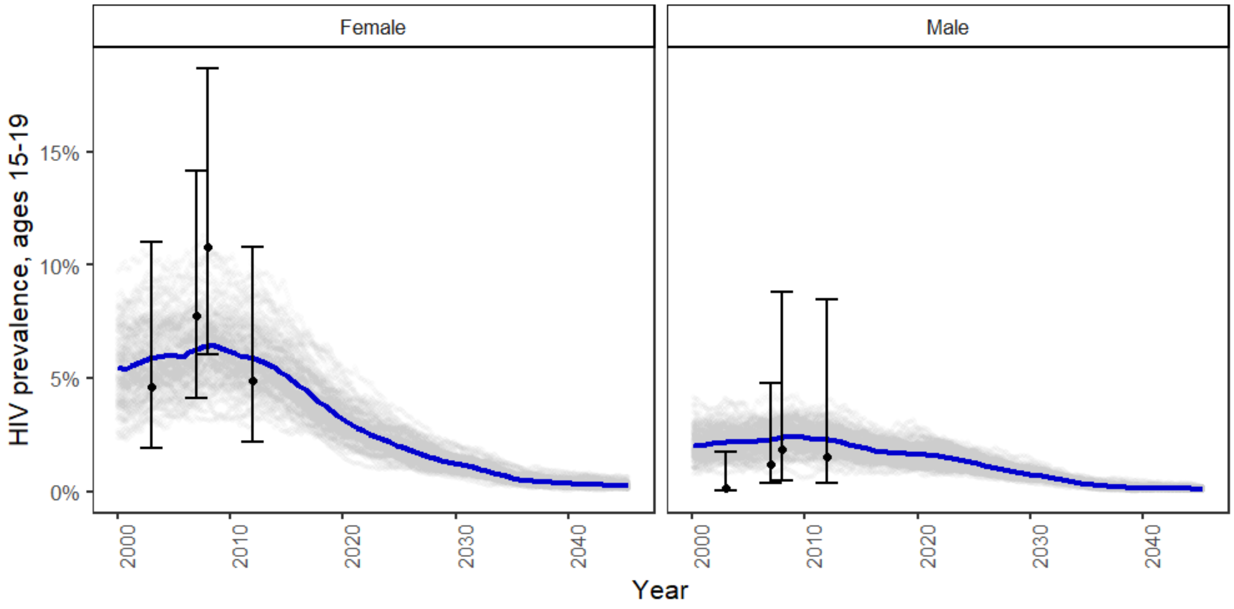

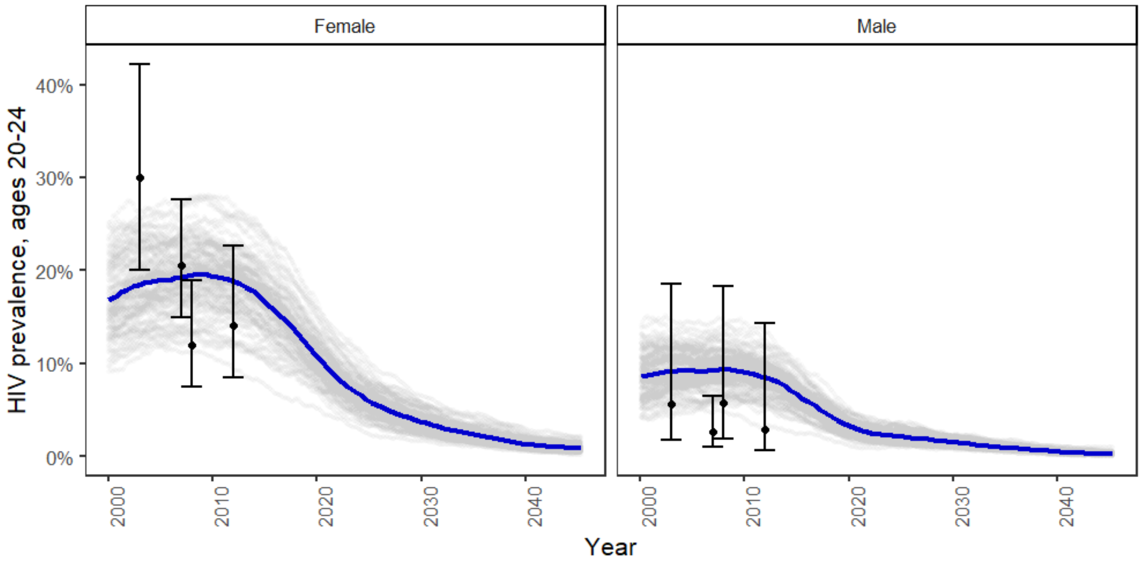
Female
Male
HIV prevalence, ages 20-24
40%
30%
20%
10%
0%
2000
2010
2020
2030
2040
Year

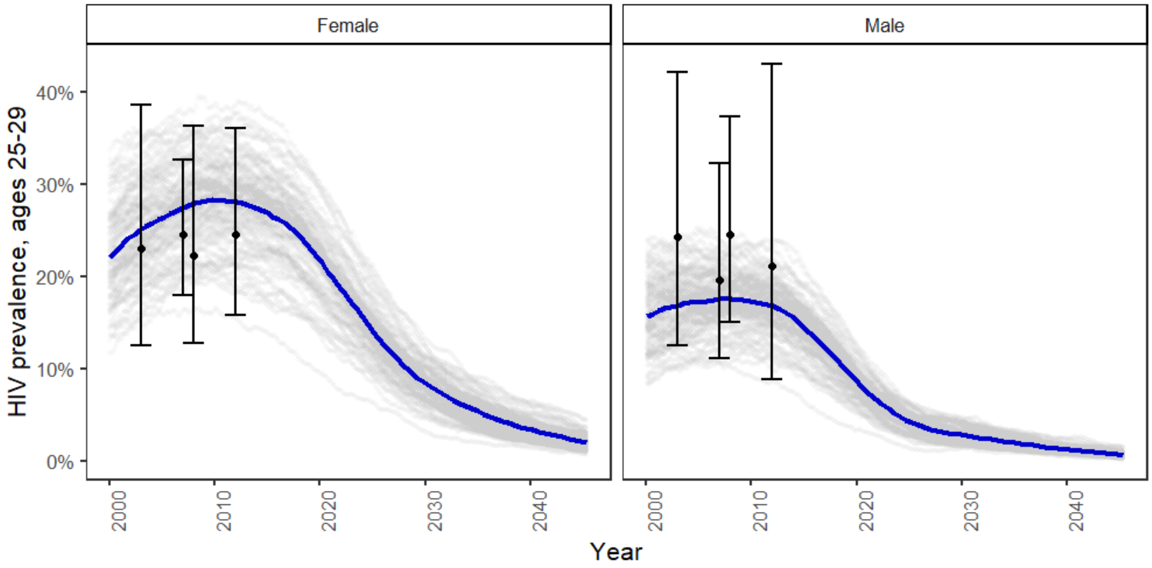
Female
Male
HIV prevalence, ages 25-29
40%
30%
20%
10%
0%
2000
2010
2020
2030
2040
Year

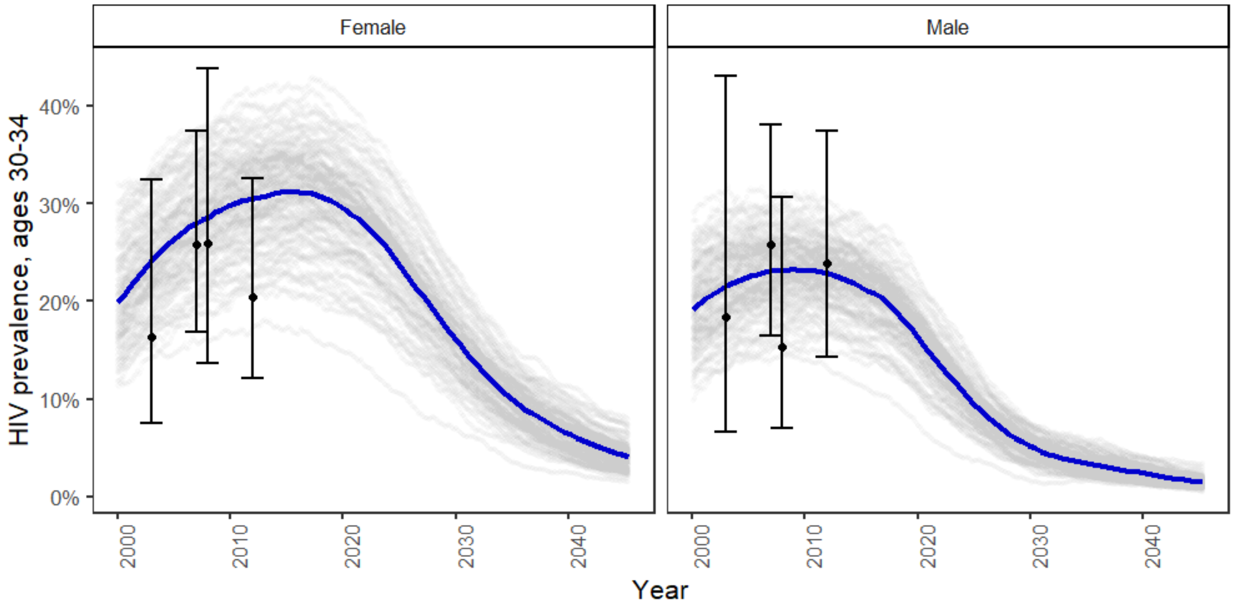
Female
Male
HIV prevalence, ages 30-34
0%
10%
20%
30%
40%
2000
2010
2020
2030
2040
Year

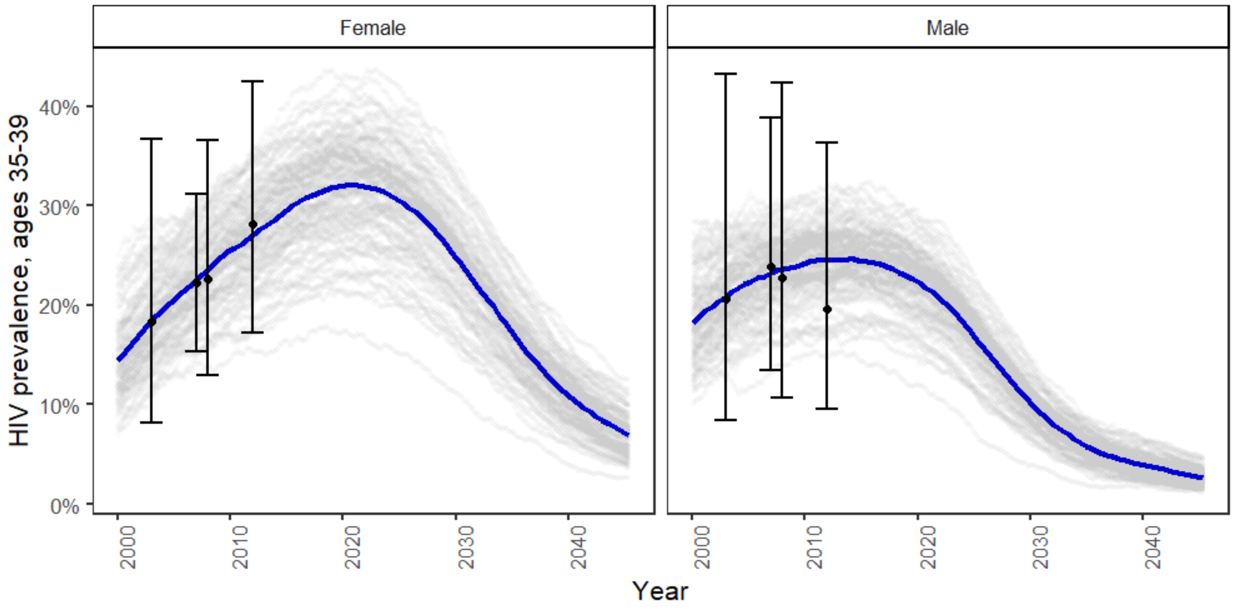
Female
Male
HIV prevalence, ages 35-39
0%
10%
20%
30%
40%
2000
2010
2020
2030
2040
Year

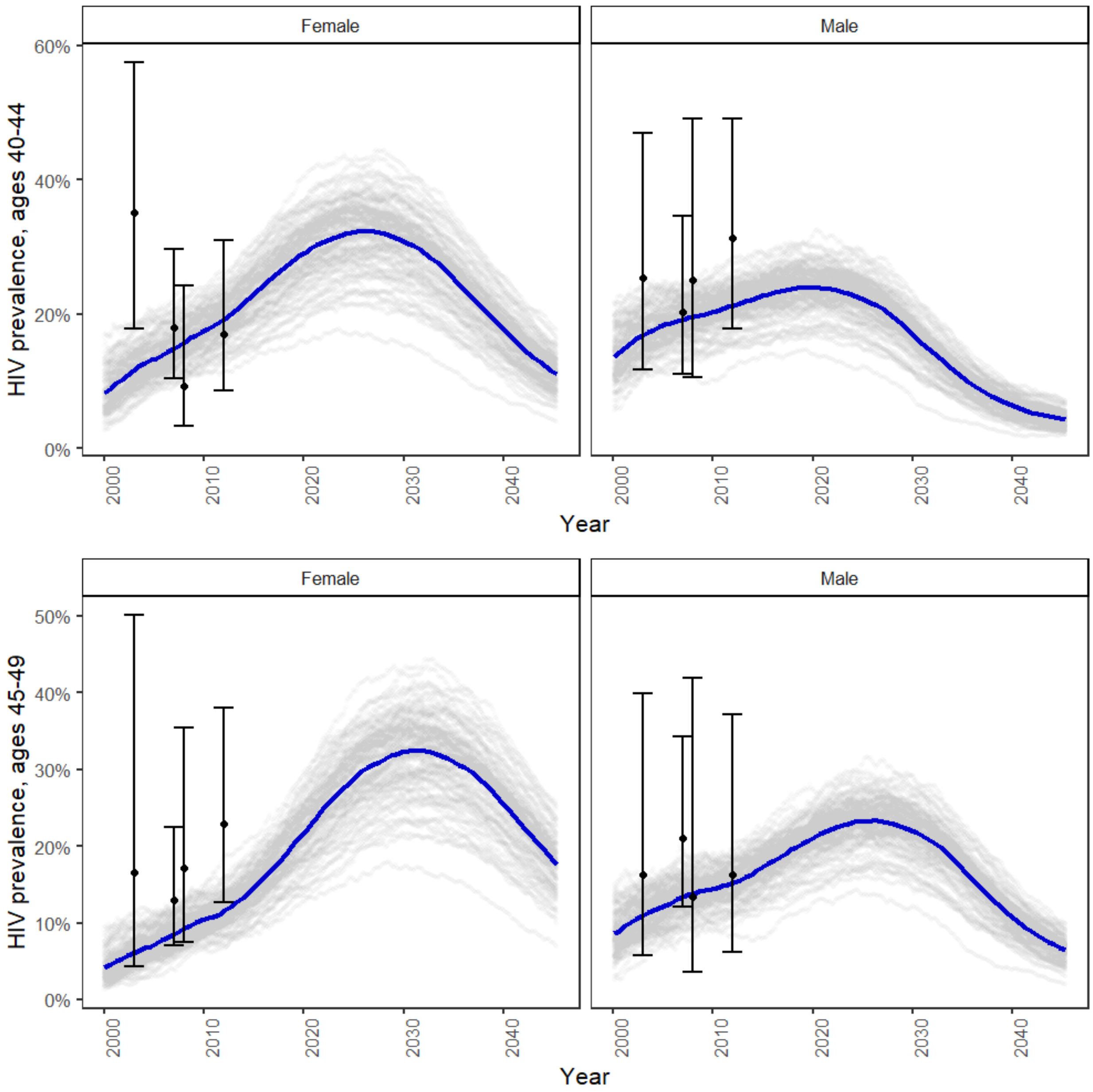

* Grey curves refer to the HIV prevalence from each individual simulation; the error bars refer to the empirical estimates and 95% confidence intervals for HIV prevalence obtained from Kenya Demographic and Health Surveys and Kenya AIDS Indicator Surveys.

Model Overview and Parameters

Model initialization

Our simulations begin prior to the start of infection in the year 1960 to allow sufficient time for the epidemic to burn-in. During this time, individuals with demographic properties specified in the demographics file begin forming relationships; relationship formation rates for each gender and relationship type are updated daily using a relationship flow algorithm. Adjustment of pair formation entry rates is terminated at a specific timepoint (e.g., 1975) and the rates are fixed at that value for the remainder of the simulation. The age profile of the population is initialized using demographic data including population age distribution or age-specific fertility rates. A prior analysis of our model evaluated the age/sex pairings, partnership length and other sexual network characteristics and confirmed that these outputs reached equilibrium within 20 years, prior to introduction of HIV into the model: https://ieeexplore.ieee.org/abstract/document/6426573.(25) HIV infections are seeded in 1980 and affect a certain proportion of the population based on the age and gender distribution reported from historical data. ART intervention is introduced in the year 2012. Eligible individuals enroll in ART based on historical eligibility criteria based on CD4 count, which changes over time based on WHO guidelines for ART initiation until the implementation of universal ART, which is assumed to remain the same until the end of the simulations (year 2050). Calibration and validation processes are performed to refine the initialization and ensure that the model aligns with observed HIV dynamics in the target population.(26, 27)

The following provides a more detailed description of the model initialization process: https://docs.idmod.org/projects/emod-hiv/en/latest/sti-model-relationships.html.(26)

Modelled time step

Our model implemented a monthly time step, aggregating events and changes over the course of each month. Monthly updated information can then be ascertained regarding a range of activities and occurrences, including sexual mixing, relationship formation, stages of HIV infection, HIV testing and its results, and PrEP initiation and discontinuation. If an individual acquires HIV and receives an HIV test in the same time step they will test HIV-negative. A limitation of the model is that we cannot ascertain when during the timestep an HIV infection occurred.

PrEP cascade

Individuals who have at least one sexual partner and who are not known to be HIV positive are given a 75% probability of undergoing HIV testing to initiate PrEP. Those who test HIV negative are assumed to complete a 30 day course of PrEP and have a 75% probability of return for a refill visit. HIV testing occurs at each PrEP continuation visit before a refill is dispensed and individuals must screen HIV-negative to receive PrEP. PrEP discontinuation occurred if individuals are lost to follow-up or no longer met the eligibility criteria (e.g., age >= 50, termination of all partnerships, or tested positive for HIV). Individuals who stopped PrEP can re-start at any time if they meet the eligibility criteria (i.e. start a new partnership) and are given the same probability of initiating PrEP as those who are PrEP-naïve.

Figure S2. EMOD-HIV PrEP cascade

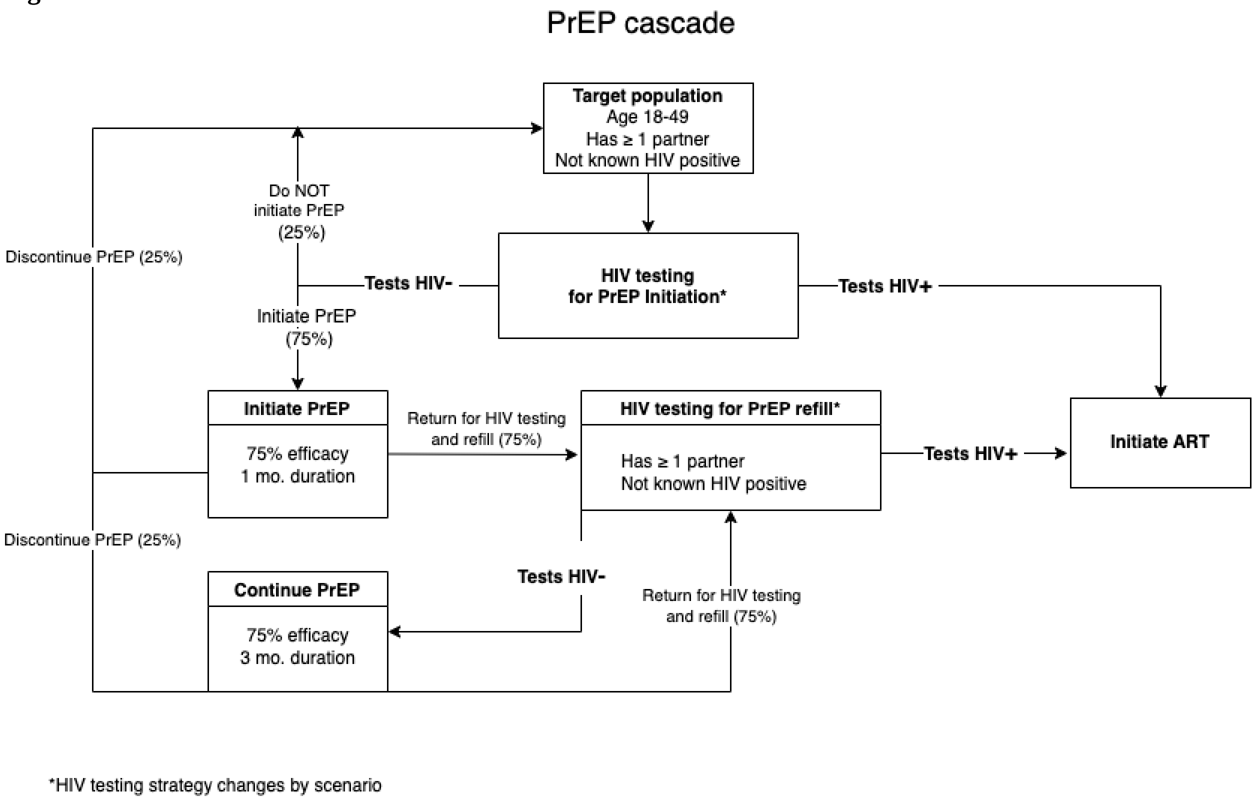

Scaling factor
We applied scaling factors of approximately 60 to each run to increase the size of our modeled population and outcomes to reflect the actual population size in western Kenya. To determine the scaling factor for each simulation, we divided the size of the Kenya 2019 census population by the size of the population in 2019 and multiplied this ratio by the modeled population and outcomes. The size of the scaling factor varied slightly depending on model run due to stochastic variability in modeled population size.

HIV testing
Background facility-based HIV testing is incorporated into the model as part of the HIV care cascade. Individuals undergo HIV testing at based at rates based on empiric data on testing coverage in the past 12 months from Kenya. In addition to the background facility-based HIV testing, we also distributed HIV testing to individuals as part of PrEP implementation. Individuals who are not known to be HIV-positive and have at least one sexual partner can receive HIV testing and subsequent PrEP provision upon testing HIV-negative. HIV testing is provided every 3 months thereafter to individuals seeking to refill PrEP in line with WHO guidelines for PrEP provision.

Model calibration
The model displays close fits to overall HIV prevalence across age groups. Additionally, the majority of the modeled age-specific HIV prevalence falls within the 95% confidence interval of empirical estimates. The model tends to underestimate HIV prevalence among the two oldest age groups for both males and females (age 40-44, 45-49). Among females, the model's estimates align well with the empiric data for the youngest age groups (age 15-19, 20-24), but tends to overestimate HIV prevalence among those aged 25-29 and 30-34. For males, the model tends to overestimate HIV prevalence for the two youngest age groups and underestimate the 25-29 age group. There is considerable uncertainty in empiric age specific estimates of HIV prevalence.

Community-based PrEP provision

We assume that individuals receiving PrEP in community-based locations obtain all PrEP associated services (e.g., HIV testing, PrEP drugs and user support) in these settings. We utilized cost estimates from a pilot study of pharmacy-based PrEP provision. Briefly, the care pathway for pharmacy-based PrEP was assumed to follow the steps observed in clinical trials. First, pharmacy providers screen interested clients for HIV risk and PrEP contraindications.(28) Providers then counsel eligible clients regarding PrEP use and safety. Next, clients complete HIV testing. The testing modality varies by scenario: in HIVST scenarios, clients complete HIVST in private back rooms of the pharmacy. In provider RDT scenarios, providers conduct HIV testing. Those who test HIV-negative are dispensed PrEP and those testing HIV-positive are referred to health care clinics for confirmatory testing and treatment. Pharmacy providers dispense a 1-month PrEP supply at initiation and a 3-month supply thereafter. At subsequent visits, clients completed a self-administered risk and PrEP safety assessment and HIV testing. Those who are eligible can obtain a PrEP refill. A remote clinician is available to pharmacists consultation as needed if medical questions arise.(29) We include costs for all aspects of the care pathway in the microcosting that informed our cost inputs for each PrEP scenario. We also assumed PrEP associated costs including HIVST and PrEP drugs would be borne by the Kenya Ministry of Health. Future studies are needed to examine other cost-sharing models, including client user fees and incentives provided to pharmacies/community distributors.

Several studies have demonstrated that trained existing pharmacy staff to provide PrEP can be a feasible, acceptable and efficient strategy, particularly in reaching those without regular access to health facilities.(30) A pilot study of pharmacy-delivered PrEP in Kenya found that most pharmacy providers are willing to provide PrEP and >80% of clients were willing to pay for PrEP services (median: $3.30 USD/visit, IQR $1.60 – $4.10).(31) Previously evaluated models of pharmacy PrEP have charged clients a nominal fee to cover provider time (e.g., 300 schillings) while others have paid the pharmacies a set monthly amount for providing PrEP.

HIV testing for PrEP initiation

We assume HIV testing occurs prior to PrEP initiation and at each refill visit before PrEP drugs are dispensed in the community. In our PrEP scenarios, model results demonstrate that among persons initiating PrEP, the average number of initiations per year is 0.35. The average person-time on PrEP is approximately 3 months and persons are tested for HIV at their one month refill visit. Therefore individuals undergo and average of 2 tests per PrEP initiation, resulting in an average number of HIV tests per year as 0.7 (0.35*2) per person taking PrEP.

Table S9a. Model parameters. Select model parameters used to fit the EMOD-HIV transmission model to survey data on prevalence and ART coverage from Kenya. Median and interquartile ranges (IQRs) reported for all dynamic parameters used in the calibration process from 100 best-fitting parameter sets. [†]

Parameter	Parameter Description	Fitted median	(IQR)
ARTLinkMax	Maximum probability of linkage to ART	0.999	(0.978, 1.000)
ARTLinkMid	Year of ART linkage (given eligibility), that is, time of the inflection point in the sigmoid trend.	2003.008	(2002.920, 2003.837)
AcuteDurationMonths	The time since infection, in months, over which the Acute_Stage_Infectivity_Multiplier is applied to coital acts occurring in that time period.	1.000	(1.000, 1.331)
CircumcisionReducedAcquire	The reduction of susceptibility to STI by voluntary male medical circumcision (VMMC).	0.600	(0.598, 0.600)
Homa_BayInfrmlCondomsMax	Maximum rate of condom use in informal relationships in Homa Bay	0.231	(0.230, 0.233)
Homa_BayLOWRisk	Proportion of the population that is low-risk in Homa Bay	0.557	(0.549, 0.559)
Homa_BayTrnsCondomsMax	Maximum rate of condom use in transitory relationships in Homa Bay	0.244	(0.232, 0.245)
InfmrlFormRate	Informal relationship formation rate	0.000	(0.000, 0.000)
InfrmlCondomMid	Year midpoint of logistic scale-up of condom use in informal relationships	1993.007	(1991.629, 1998.680)
InfrmlCondomRate	Rate of logistic scale-up of condom use in informal relationships	2.661	(1.953, 2.909)
InfrmlCondomsMax	Maximum rate of condom use in informal relationships	0.216	(0.215, 0.217)
InfrmlDurHet	Heterogeneity in duration of informal relationships	0.750	(0.750, 0.750)
KisiiInfrmlCondomsMax	Maximum rate of condom use in informal relationships in Kisii	0.231	(0.226, 0.232)
KisiiLOWRisk	Proportion of the population that is low-risk in Kisii	0.938	(0.935, 0.939)
KisiiTrnsCondomsMax	Maximum rate of condom use in transitory relationships in Kisii	0.375	(0.369, 0.376)
KisumuInfrmlCondomsMax	Maximum rate of condom use in informal relationships in Kisumu	0.182	(0.181, 0.183)
KisumuLOWRisk	Proportion of the population that is low-risk in Kisumu	0.764	(0.762, 0.765)
KisumuTrnsCondomsMax	Maximum rate of condom use in transitory relationships in Kisumu	0.338	(0.337, 0.347)
LogBaseInfectivity	The probability of transmission when none of the transmission multipliers apply to a particular coital act.	0.002	(0.002, 0.002)
MaleToFemaleOld	Male-to-female relative risk of infection among older individuals	2.012	(1.506, 2.132)
MaleToFemaleYoung	Male-to-female relative risk of infection among young individuals	1.116	(1.000, 1.242)
MaxInfrmlFLOW	Maximum number of informal relationships among low-risk females	1.311	(1.255, 1.315)
MaxInfrmlFMED	Maximum number of informal relationships among medium-risk females	2.050	(2.002, 2.351)
MaxInfrmlMLOW	Maximum number of informal relationships among low-risk males	1.165	(1.159, 1.203)
MaxInfrmlMMED	Maximum number of informal relationships among medium-risk males	2.530	(2.442, 2.735)
MaxMrtlFMED	Maximum number of marital relationship among medium-risk females	1.139	(1.136, 1.159)
MaxMrtlMMED	Maximum number of marital relationship among medium-risk males	1.302	(1.268, 1.316)
MaxTrnsFLOW	Maximum number of transitory relationships among low-risk females	1.599	(1.580, 1.625)

Parameter	Parameter Description	Fitted median	(IQR)
MaxTrnsFMED	Maximum number of transitory relationships among medium-risk females	2.943	(2.891, 3.000)
MaxTrnsMLOW	Maximum number of transitory relationships among low-risk males	1.599	(1.588, 1.677)
MaxTrnsMMED	Maximum number of transitory relationships among medium-risk males	2.557	(2.475, 2.879)
MigoriInfrmlCondomsMax	Maximum rate of condom use in informal relationships in Migori	0.186	(0.185, 0.189)
MigoriLOWRisk	Proportion of the population that is low-risk in Migori	0.795	(0.793, 0.797)
MigoriTrnsCondomsMax	Maximum rate of condom use in transitory relationships in Migori	0.249	(0.248, 0.257)
MrtlCondomMax	Maximum rate of condom use in marital relationships	0.192	(0.191, 0.193)
MrtlCondomMid	Year midpoint of logistic scale-up of condom use in marital relationships	2003.131	(1999.017, 2005.000)
MrtlCondomRate	Rate of logistic scale-up of condom use in marital relationships	2.971	(2.595, 3.000)
MrtlFormRate	Marital relationship formation rate	0.000	(0.000, 0.000)
NyamiraInfrmlCondomsMax	Maximum rate of condom use in informal relationships in Nyamira	0.096	(0.093, 0.097)
NyamiraLOWRisk	Proportion of the population that is low-risk in Nyamira	0.902	(0.900, 0.910)
NyamiraTrnsCondomsMax	Maximum rate of condom use in transitory relationships in Nyamira	0.309	(0.304, 0.313)
PrExInfrmlFemLOW	Probability of potential for extra-relational informal relationship among low-risk females	0.366	(0.364, 0.371)
PrExInfrmlFemMED	Probability of potential for extra-relational informal relationship among medium-risk females	0.401	(0.401, 0.407)
PrExInfrmlMaleLOW	Probability of potential for extra-relational informal relationship among low-risk males	0.244	(0.222, 0.249)
PrExInfrmlMaleMED	Probability of potential for extra-relational informal relationship among medium-risk males	0.382	(0.379, 0.389)
PrExTrnsFemLOW	Probability of potential for extra-relational transitory relationship among low-risk females	0.033	(0.033, 0.033)
PrExTrnsFemMED	Probability of potential for extra-relational transitory relationship among medium-risk females	0.460	(0.450, 0.468)
PrExTrnsMaleLOW	Probability of potential for extra-relational transitory relationship among low-risk males	0.330	(0.328, 0.332)
PrExTrnsMaleMED	Probability of potential for extra-relational transitory relationship among medium-risk males	0.593	(0.589, 0.611)
PreARTLinkMax	Maximum probability of linkage to pre-ART care	0.715	(0.708, 0.737)
PreARTLinkMid	Year midpoint of logistic scale-up of pre-ART linkage	1996.709	(1995.685, 1998.896)
PreARTLinkMin	Minimum probability of linkage to pre-ART care	0.435	(0.416, 0.442)
RiskAssortivity	Risk assortivity	0.663	(0.648, 0.673)
SeedYrHigh	Seed year	1986.562	(1982.000, 1988.000)
SexualDebutAgeFemaleWeibullHeterogeneity	Heterogeneity parameter of Weibull distribution of female age of sexual debut	0.086	(0.083, 0.086)
SexualDebutAgeFemaleWeibullScale	Scale parameter of Weibull distribution of female age of sexual debut	16.013	(15.540, 16.038)
SexualDebutAgeMaleWeibullHeterogeneity	Heterogeneity parameter of Weibull distribution of male age of sexual debut	0.040	(0.040, 0.040)
SexualDebutAgeMaleWeibullScale	Scale parameter of Weibull distribution of male age of sexual debut	15.708	(15.155, 16.090)
SiayaInfrmlCondomsMax	Maximum rate of condom use in informal relationships in Siaya	0.160	(0.148, 0.161)
SiayaLOWRisk	Proportion of the population that is low-risk in Siaya	0.729	(0.722, 0.731)
SiayaTrnsCondomsMax	Maximum rate of condom use in transitory relationships in Siaya	0.300	(0.293, 0.304)

Parameter	Parameter Description	Fitted median	(IQR)
TrnsCondomMax	Maximum rate of condom use in transitory relationships	0.243	(0.242, 0.253)
TrnsCondomMid	Year midpoint of logistic scale-up of condom use in transitory relationships	1997.960	(1996.962, 1999.273)
TrnsCondomRate	Rate of logistic scale-up of condom use in transitory relationships	0.999	(0.978, 1.000)
TrnsFormRate	Transitory relationship formation rate	2003.008	(2002.920, 2003.837)

† We utilized previously published version of EMOD-HIV and recalibrated with most recent data.(26,27) The only modifications to the default parameterization outside of PrEP implementation and HIV test sensitivity (described in the main manuscript text) are detailed in Table S9 above. A full description of all parameters and references available is at: https://docs.idmod.org/projects/emod-hiv/en/latest/parameter-overview.html and elsewhere.(26,27)

Table S9b. Cost parameter calculations[¥]

Cost Parameter	Estimate (USD)	Year[†]	Data source	Calculation details, notes
Annual health care costs (among those not on ART)				
HIV-positive CD4 Under 200	110.30	2021	(32)	Adjusted for inflation and GDP/capita ratio<br><br>Step 1: Adjust South Africa value in 2012 USD for inflation to be in 2021 USD ($374.08 = $167*2.24)<br>● Cost of health care use, CD4 count <200 cells per μL, not in HIV care (per person-year) in South Africa= $167<br>● USD Inflation Rate between time of costing (2012) and 2021: 2.24 =4.7/2.1<br>Step 2: Adjust South Africa 2021 USD value by multiplying by the Kenya GDP/cap ratio ($374.08*0.295)<br>● South Africa 2021 GDP per capita in $USD = 7,055<br>● Kenya 2021 GDP per capita in $USD = 2,082<br>● Kenya GDP/ ZA GDP ratio adjustment: (2,082/7,055)= 0.295
HIV-positive CD4 200 To 349	30.38	2021	(32)	Adjusted for inflation and GDP/capita ratio<br><br>Step 1: Adjust South Africa value in 2012 USD for inflation to be in 2021 USD ($103.04 = $46*2.24)<br>● Cost of health care use, CD4 count 200-349 cells per μL, not in HIV care (per person-year) in South Africa= $46<br>● USD Inflation Rate between time of costing (2012) and 2021: 2.24 =4.7/2.1<br>Step 2: Adjust South Africa 2021 USD value by multiplying by the Kenya GDP/cap ratio ($103.04*0.295)<br>● South Africa 2021 GDP per capita in $USD = 7,055<br>● Kenya 2021 GDP per capita in $USD = 2,082<br>● Kenya GDP/ ZA GDP ratio adjustment: (2,082/7,055)= 0.295
HIV-positive CD4 350 Plus	8.59	2021	(32)	Adjusted for inflation and GDP/capita ratio<br><br>Step 1: Adjust South Africa value in 2012 USD for inflation to be in 2021 USD ($29.1 = $13*2.24)<br>● Cost of health care use, CD4 count >350 cells per μL, not in HIV care (per person-year) in South Africa= $13<br>● USD Inflation Rate between time of costing (2012) and 2021: 2.24 =4.7/2.1<br>Step 2: Adjust South Africa 2021 USD value by multiplying by the Kenya GDP/cap ratio ($29.1*0.295)<br>● South Africa 2021 GDP per capita in $USD = 7,055<br>● Kenya 2021 GDP per capita in $USD = 2,082<br>● Kenya GDP/ ZA GDP ratio adjustment: (2,082/7,055)= 0.295
Annual ART provision costs	140.89	2021/ 2016	(33-35)	Includes 20% mark up on ART drug costs to account for supply chain (2021 USD) (REF). ART delivery costs are from an in-country micro-costing study and include lab tests and staff encounters (2016 USD).

				($140.89=57.89+83)<br><br>Step 1: Calculate annual ART cost accounting for 20% supply chain cost mark-up ($57.89= $48.24*1.2)<br>● Monthly ART cost (per pack, 30 for Dolutegravir/Lamivudine/Tenofovir 50/300/300mg tablet)= $4.02 (31)<br>● Annual ART cost= $48.24= $4.02*12<br>● 20% supply chain cost mark-up (29)= *1.2<br>Step 2: Calculate total cost of ART delivery ($83=45+38)<br>● $45 lab costs (30)<br>● $38 staff encounters (30)
End of life care	105.68	2021	(32)	Adjusted for inflation and GDP/capita ratio<br><br>Step 1: Adjust South Africa value in 2012 USD for inflation to be in 2021 USD ($102.95= $46*2.24)<br>● USD Inflation Rate between time of costing (2012) and 2021: 2.24 =4.7/2.1<br>Step 2: Adjust South Africa 2021 USD value by multiplying by the Kenya GDP/cap ratio ($102.95*0.295):<br>● South Africa 2021 GDP per capita in $USD = 7,055<br>● Kenya 2021 GDP per capita in $USD = 2,082<br>● Kenya GDP/ ZA GDP ratio adjustment: (2,082/7,055)= 0.295
PrEP provision (monthly)	10.66	2019	(36,37)	Data from in-country micro-costing studies (removing lab testing costs)<br><br>Chose higher and thus more conservative estimate (33): ($10.66= $13.5-$2.84)<br>● Cost of PrEP per client per month using HIVST= $13.5<br>● Cost of lab testing for PrEP= $2.84
Facility-based HIV-positive test	3.68	2017	(38)	Data from micro-costing study. Inputs include screening test kit, other supply costs, and personnel costs.
Facility-based HIV-negative test	2.64	2017	(38)	Data from micro-costing study. Inputs include screening test kit, other supply costs, and personnel costs.
HIV Ab RDT	1.21	2021	(36,39)	Includes HIV RDT test cost plus pharmacist counseling time estimated from an in-country micro-costing study of pharmacy-based PrEP implementation.<br><br>($1.21= $0.90 + $0.31)<br>● Cost of RDT test (upper bound)= $0.90 (35)<br>● Cost for counseling (counseling for PrEP initiation = 7.3 minutes)= $0.31 (32)
Blood-based HIVST	5	2021	Expert opinion	
Oral fluid HIVST	3	2021	Expert opinion	
NAT test	22	2021	Expert opinion	

¥PrEP: pre-exposure prophylaxis, NAT: nucleic acid test, ART: antiretroviral therapy. Incident infection refers to those who seroconvert while taking PrEP. All HIV test, ART and PrEP provision costs include personnel, overhead, and supplies.
†Inflation adjustment: Adjustment using data from the World Bank (% inflation, consumer prices) (40) applied only if reference provides cost estimates for years prior to 2016.

Figure S3. Model predicted population growth by sex

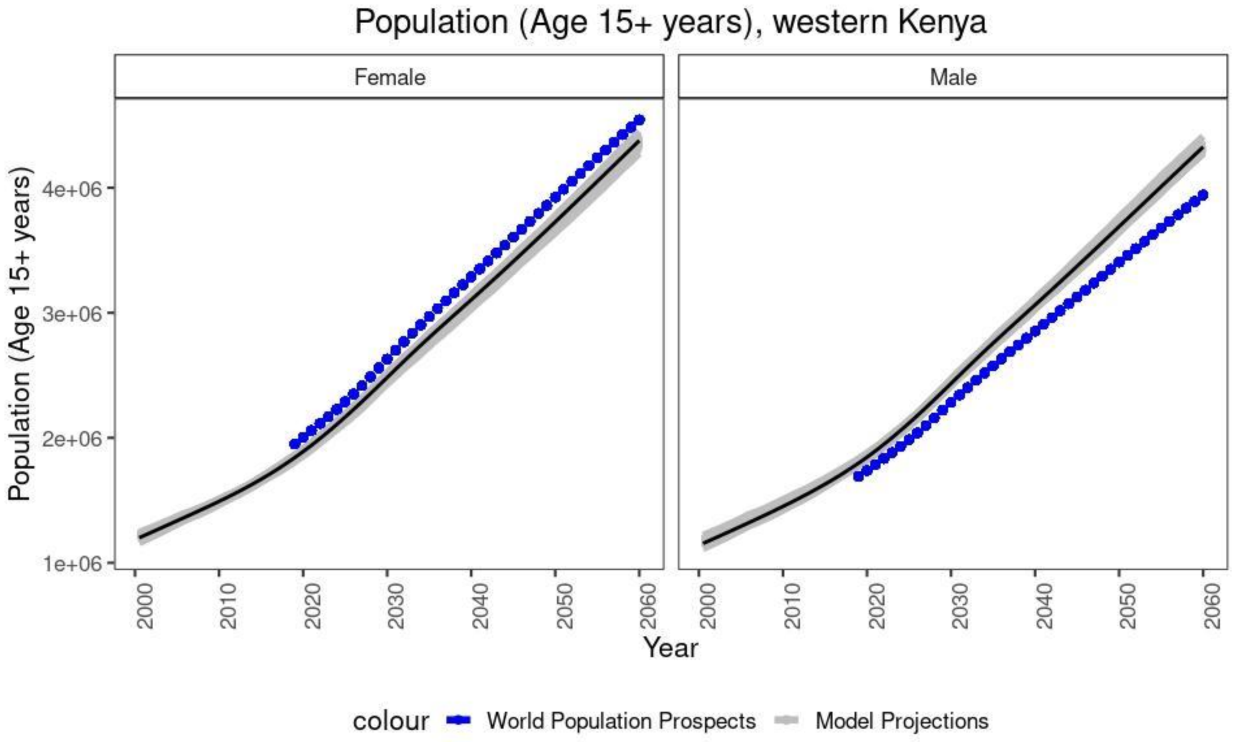

Other Supplemental Tables and Figures

Table S10. Budget impact of 5-year PrEP scale-up by HIV testing modality assuming a population of 3.1 million individuals.

	No PrEP (baseline)	Oral HIVST	Blood HIVST	Ab RDT	NAT
**Budget Impact**	$73.3	$258.5	$277.1	$241.6	$436.3
(2021 $USD, millions)	($62.3 - $82.5)	($246.5 - $269.8)	($265.0 - $289.4)	($229.9 - $252.8	($414.6 - $455.2)
% of baseline	-	353%	378%	330%	595%
**Component cost**					
HIV testing	$32.2	$85.2	$104.0	$68.4	$263.2
	($30.9 - $33.6)	($80.9 - $89.9)	($98.6 - $109.8)	($65.3 - $72.4)	($247.0 - $279.3)
HIV-related illness	$15.3	$11,7	$11.6	$11.6	$11.6
	($10.7 - $19.4)	($8.2 - $14.9)	($8.2 - $14.5)	($8.3 - $14.6)	($8.0- $14.9)
ART provision	$25.9	$26.2	$26.2	$26.2	$26.2
	($18.3 - $33.2)	($18.5 - $433.6)	($18.5 - $33.5)	($18.5 - $33.6)	($18.5 - $33.6)
PrEP provision	$0.0	$135.4	$135.3	$135.4	$135.3
	($0.0 - $0.0)	($126.1 - $144.7)	($124.7 - $144.2)	($124.7 - $144.5)	($125.8 - $144.2)

Table S11. Details for modelled PrEP impact and resistance outcomes by HIV test scenario over 20 years among individuals age 18-49 years

	No PrEP (baseline)	Oral HIVST	Blood HIVST	Ab RDT	NAT
**HIV infections**	619,042<br>(423,286 - 789,257)	545,434<br>(380,531 - 695,641)	545,554<br>(383,818 - 689,475)	545,068<br>(382,290 - 693,820)	544,857<br>(380,920 - 697,999)
**HIV deaths**	35,471<br>(25,339 - 45,717)	29,251<br>(20,263 - 36,936)	29,226<br>(21,288 - 37,922)	29,101<br>(20,198 - 36,771)	29,293<br>(21,028 - 37,949)
**Individuals with HIV inappropriately initiated on PrEP**					
**Acute**	-	5,650<br>(955 - 15,147)	5,172<br>(945 - 14,980)	2,214<br>(774 - 5,008)	1,312<br>(635 - 2,103)
**Chronic**	-	11,942<br>(3,661 - 20,439)	6,842<br>(764 - 14,254)	1,739<br>(77 - 3,571)	132<br>(0 - 460)
**Individuals w/ HIV and NRTI resistance**	185,713<br>(126,986 - 236,777)	167,877<br>(117,384 - 215,473)	166,913<br>(117,891 - 212,292)	164,946<br>(115,671 - 210,432)	164,318<br>(114,739 - 210,745)
Acute	-	1,865<br>(315 - 4,999)	1,707<br>(312 - 4,944)	731<br>(256 - 1,653)	433<br>(210 - 694)
Chronic	-	1,971<br>(604 - 3,373)	1,129<br>(127 - 2,352)	287<br>(13 - 590)	22<br>(0 - 76)
Incident infection	-	412<br>(227 - 589)	412<br>(249 - 596)	408<br>(248 - 577)	406<br>(226 - 579)
Background	185,713<br>(126,986 - 236,777)	163,631<br>(114,160 - 208,693)	163,667<br>(115,146 - 206,843)	163,521<br>(114,687 - 208,146)	163,458<br>(114,276 - 209,400)

Table S12. Sensitivity analysis of modelled PrEP impact and resistance outcomes by test scenario over 20 years among individuals age 18-49 years*

	Oral HIVST		Blood HIVST	
	**Pessimistic**	**Very pessimistic**	**Pessimistic**	**Very pessimistic**
**HIV infections**	545,985<br>(380,531 - 695,641)	545,695<br>(381,753 - 693,822)	545,716<br>(380,622 - 696,429)	545,714<br>(381,046 - 697,133)
**Infections averted** (%)	53.9<br>(49.3 - 59.2)	54.1<br>(49.5 - 58.7)	53.9<br>(48.1- 58.6)	54.0<br>(48.7 - 58.7)
**HIV deaths**	29,407<br>(20,481 - 38,260)	29,790<br>(20,182 - 38,563)	29,391<br>(20,454 - 37,474)	29,633<br>(19,594 - 38,128)
**Deaths averted** (%)	17.0<br>(8.5 - 25.5)	15.9<br>(8.0 - 24.3)	17.0<br>(9.4 - 25.1)	16.4<br>(7.3 - 26.0)
**PrEP initiations** (millions)	42.8<br>(40.2 - 45.2)	42.8<br>(40.2 - 45.1)	42.8<br>(40.2 - 44.9)	42.8<br>(40.4 - 45.3)
**Individuals with HIV inappropriately initiated on PrEP**				
**Acute**	5,718<br>(832 - 16,527)	5,778<br>(949 - 15,770)	5,348<br>(828 - 14,500)	5,755<br>(940 - 15, 756)
**Chronic**	23,698<br>(11,649 - 33,539)	28,043<br>(15,081 - 39,228)	19,378<br>(9,485 - 27,865)	24,391<br>(12,355 - 34,970)
**Individuals with incident HIV while on PrEP**	14,785<br>(8,315 - 22,002)	14,728<br>(8,418 - 21,092)	14,741<br>(8,952 - 21,048)	14,643<br>(8,499 - 20,330)
**Average HIV person-months on PrEP**	3.1<br>(2.9 - 3.2)	3.2<br>(3.0 - 3.4)	2.9<br>(2.8 - 3.0)	3.1<br>(3.0 - 3.2)
**Individuals with HIV and NRTI resistance**	170,007<br>(119,029 - 219,119)	170,654<br>(119,440 - 218,930)	169,090<br>(118,205 - 217,575)	170,048<br>(118,361 - 219,715)
Acute	5,718<br>(832 - 16,527)	5,777<br>(949 - 15,770)	5,348<br>(829 - 14,500)	5,755<br>(940, 15,756)
Chronic	23,698<br>(11,649 - 33,539)	28,043<br>(15,081 - 39,228)	19,378<br>(9,485 - 27,865)	24,391<br>(12,355 - 34,970)
Incident infection	14,785<br>(8,315 - 22,002)	14,728<br>(8,418 - 21,092)	14,741<br>(8,952 - 21,048)	14,643<br>(8,499 - 20,330)
Background	163,796<br>(114,247 - 209,304)	163,709<br>(114,526 - 208,147)	163,715<br>(114,187 - 208,929)	163,715<br>(114,314 - 209,140)
**HIV infections with PrEP-associated NRTI resistance** (%)	1.1<br>(0.9 - 1.5)	1.3<br>(1.0 - 1.6)	1.0<br>(0.8 - 1.3)	1.2<br>(0.9 - 1.5)
**Population prevalence of NRTI resistance** (%)	4.6<br>(3.2 - 5.9)	4.6<br>(3.2 - 5.9)	4.6<br>(3.2 - 5.9)	4.6<br>(3.2 - 5.9)

*Pessimistic and very pessimistic scenarios assumed 30% lowered sensitivity of HIV test (i.e., multiplied sensitivity by 0.7 across all time points from the base case). An additional 34 day delay in seroconversion for individuals on PrEP was further applied to the very pessimistic scenario.

Table S13a. Resistance outcomes at 50% resistance increase and 100% resistance by HIV test scenario over 20 years among individuals age 18-49 years*

	No PrEP (baseline)	Oral HIVST	Blood HIVST	Ab RDT	NAT
50% resistance increase					
**Individuals with HIV and NRTI resistance**	185,713 (126,986 - 236,777)	172,123 (120,609 - 222,219)	170,160 (120,636 - 217,742)	166,371 (116,655 - 212,718)	165,178 (115,201 - 212,016)
Acute	-	3,729 (630 - 9,997)	3,414 (624 - 9,887)	1,462 (511 - 3,306)	866 (419 - 1,388)
Chronic	-	3,941 (1,208 - 6,745)	2,258 (253 - 4,704)	574 (26 - 1,179)	44 (0 - 152)
Incident infection	-	824 (453 - 1,178)	823 (497 - 1,191)	816 (497 - 1,154)	812 (451 - 1,157)
Background	185,713 (126,986 - 236,777)	163,631 (114,160 - 208,693)	163,667 (115,146 - 206,843)	163,521 (114,687 - 208,146)	163,458 (114,276 - 209,400)
**HIV infections with PrEP- associated NRTI resistance** (%)	0.0 (0.0 - 0.0)	1.5 (1.1 - 2.1)	1.2 (0.8 - 1.8)	0.5 (0.4 - 0.7)	0.3 (0.2 - 0.4)
**Population prevalence of NRTI resistance** (%)	5.0 (3.4 - 6.4)	4.6 (3.3 - 6.0)	4.6 (3.3 - 5.9)	4.5 (3.2 - 5.7)	4.5 (3.1 - 5.7)
100% resistance					
**Individuals with HIV and NRTI resistance**	185,713 (126,986 - 236,777)	195,920 (133,315 - 251,921)	190,361 (132,847 - 242,377)	182,038 (125,954 - 235,986)	179,385 (123,948 - 231,853)
Acute	-	5,650 (955 - 15,147)	5,172 (945 - 14,980)	2,214 (774 - 5,008)	1,312 (635 - 2,103)
Chronic	-	11,942 (3,661 - 20,439)	6,842 (764 - 14,254)	1,739 (77 - 3,571)	132 (0 - 460)
Incident infection	-	14,699	14,682	14,565	14,484

		(8,079 - 21,025)	(8,872 - 21,263)	(8,863 - 20,597)	(8,043 - 20,649)
Background	185,713<br>(126,986 - 236,777)	163,631<br>(114,160 - 208,693)	163,667<br>(115,146 - 206,843)	163,521<br>(114,687 - 208,146)	163,458<br>(114,276 - 209,400)
**HIV infections with PrEP- associated NRTI resistance** (%)	0.0<br>(0.0 - 0.0)	5.9<br>(4.7 - 6.7)	4.8<br>(3.9 - 5.8)	3.3<br>(2.6 - 4.0)	2.9<br>(2.2 - 3.6)
**Population prevalence of NRTI resistance** (%)	5.0<br>(3.4 - 6.4)	5.3<br>(3.6 - 6.8)	5.1<br>(3.6 - 6.5)	4.9<br>(3.4 - 6.4)	4.8<br>(3.4 - 6.3)

*Baseline resistance probabilities for acute HIV infection, latent HIV infection, and incident HIV infection were 0.33, 0.165, and 0.028, respectively. 50% resistance increase led to resistance probabilities of 0.66, 0.33, and 0. 056 for acute, latent, and incident HIV infection, respectively. 100% resistance represents everyone inappropriately on PrEP developing resistance (e.g., resistance probability= 1.0 for acute, latent, and incident HIV infection). Increase in probability of resistance development is applied to PrEP-associated resistance only, not background resistance.

Table S13b. Sensitivity analysis of resistance outcomes at 50% resistance increase and 100% resistance by test scenario over 20 years among individuals age 18-49 years*

	Oral HIVST		Blood HIVST	
	**Pessimistic**	**Very pessimistic**	**Pessimistic**	**Very pessimistic**
50% resistance increase				
**Individuals with HIV and NRTI resistance**	176,217<br>(123,621 - 228,934)	177,600<br>(124,354 - 229,714)	174,465<br>(122,224 - 226,221)	176,382<br>(122,408 - 229,742)
Acute	3,774<br>(549 - 10,908)	3,813<br>(627 - 10,408)	3,530<br>(547 - 9,570)	3,799<br>(620 - 10,399)
Chronic	7,821<br>(3,844 - 11,068)	9,255<br>(4,977 - 12,946)	6,395<br>(3,130 - 9,196)	8,049<br>(4,077 - 11,540)
Incident infection	828<br>(466 - 1,233)	825<br>(472 - 1,182)	826<br>(502 - 1,179)	820<br>(476 - 1,139)
Background	163,796<br>(114,247 - 209,304)	163,709<br>(114,526 - 208,147)	163,715<br>(114,187 - 208,929)	163,715<br>(114,314 - 209,140)
**HIV infections with PrEP-associated NRTI resistance** (%)	2.3<br>(1.8 - 3.0)	2.5<br>(2.1 - 3.2)	2.0<br>(1.6 - 2.5)	2.3<br>(1.9 - 3.0)
**Population prevalence of NRTI resistance** (%)	4.8<br>(3.3 - 6.2)	4.8<br>(3.3 - 6.2)	4.7<br>(3.3 - 6.1)	4.8<br>(3.3 - 6.2)
100% resistance				
**Individuals with HIV and NRTI resistance**	207,995<br>(142,236 - 269,385)	212,255<br>(145,290 - 271,651)	203,181<br>(139,321 - 263,632)	208,502<br>(141,070 - 272,253)
Acute	5,718<br>(832 - 16,527)	5,777<br>(949 - 15,770)	5,348<br>(829 - 14,500)	5,755<br>(940 - 15,756)
Chronic	23,698<br>(11,649 - 33,539)	28,043<br>(15,081 - 39,228)	19,378<br>(9,485 - 27,865)	24,391<br>(12,355 - 34,970)
Incident infection	14,785<br>(8,315 - 22,002)	14,728<br>(8,418 - 21,092)	14,741<br>(8,952 - 21,048)	14,643<br>(8,499 - 20,330)
Background	163,796<br>(114,247 - 209,304)	163,709<br>(114,526 - 208,147)	163,715<br>(114,187 - 208,929)	163,715<br>(114,314 - 209,140)
**HIV infections with PrEP-associated NRTI resistance** (%)	8.0<br>(6.6 - 9.4)	8.8<br>(7.4 - 10.0)	7.2<br>(6.1 - 8.3)	8.1<br>(6.7 - 9.4)
**Population prevalence of NRTI resistance** (%)	5.6<br>(3.9 - 7.3)	5.7<br>(3.9 - 7.3)	5.5<br>(3.8 - 7.1)	5.6<br>(3.8 - 7.3)

*Baseline resistance probabilities for acute HIV infection, latent HIV infection, and incident HIV infection were 0.33, 0.165, and 0.028, respectively. 50% resistance increase led to resistance probabilities of 0.66, 0.33, and 0. 056 for acute, latent, and incident HIV infection, respectively. 100% resistance represents everyone inappropriately on PrEP developing resistance (e.g., resistance probability= 1.0 for acute, latent, and incident HIV infection). Increase in probability of resistance development is applied to PrEP-associated resistance only, not background resistance.

Figure S4. PrEP coverage over time among 18-49 years old in blood-based HIV self-testing scenario

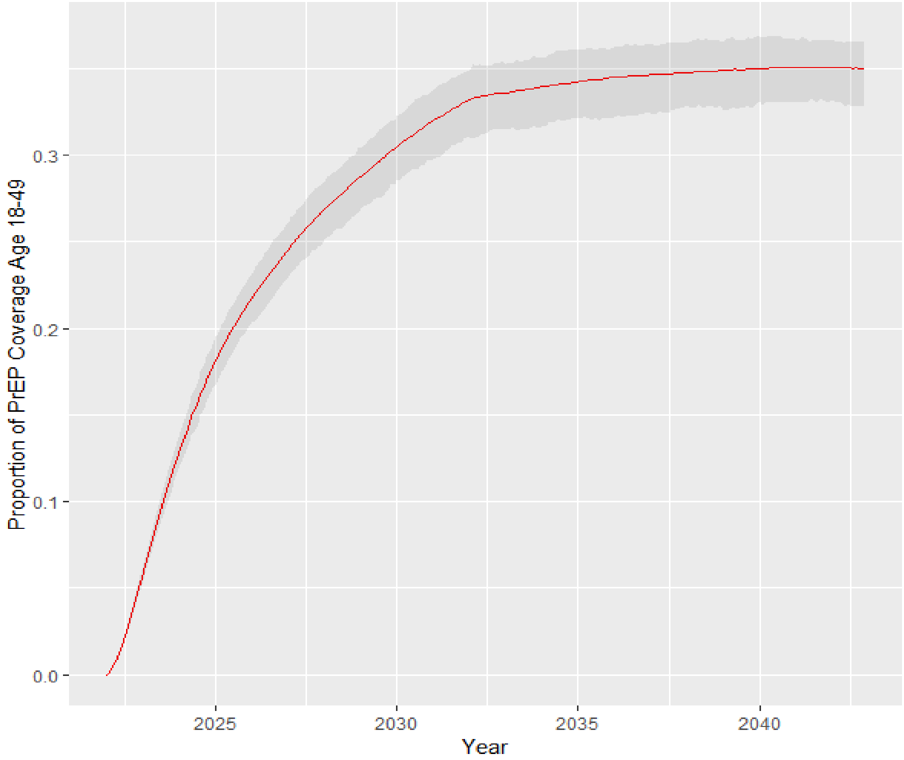

*Shaded area refers to the 95% uncertainty interval of PrEP coverage. PrEP coverage was calculated by dividing the total person-time on PrEP by the total person-time for the entire modeled population over the 20-year time horizon.

Figure S5. Proportion of HIV deaths and infection averted by scenario

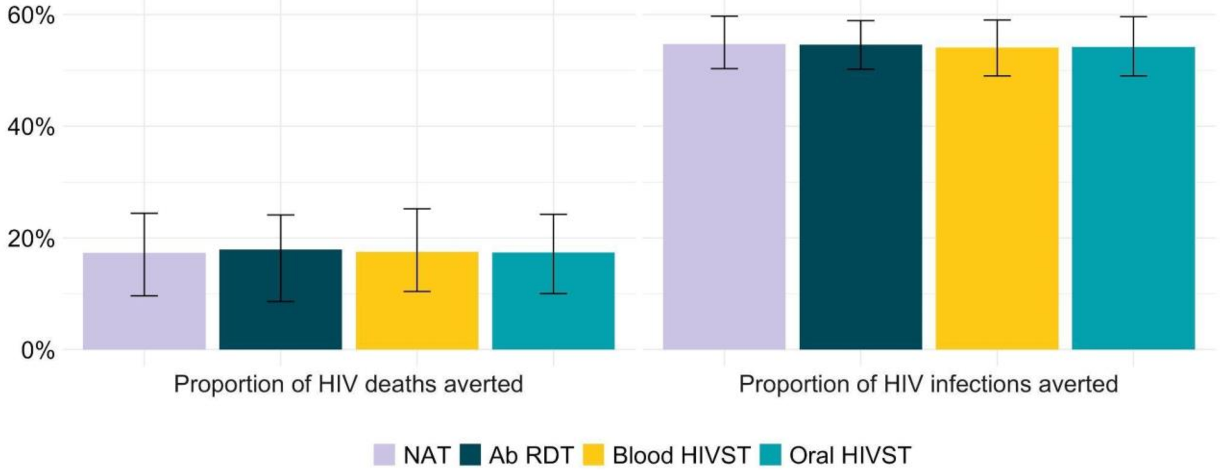

*Error bars refer to the 95% uncertainty intervals (UI) across the 100 parameter sets.

Figure S6a. Budget impact and cost components of 5-year PrEP scale-up by HIV testing modality, assuming a population of 3.1 million individuals

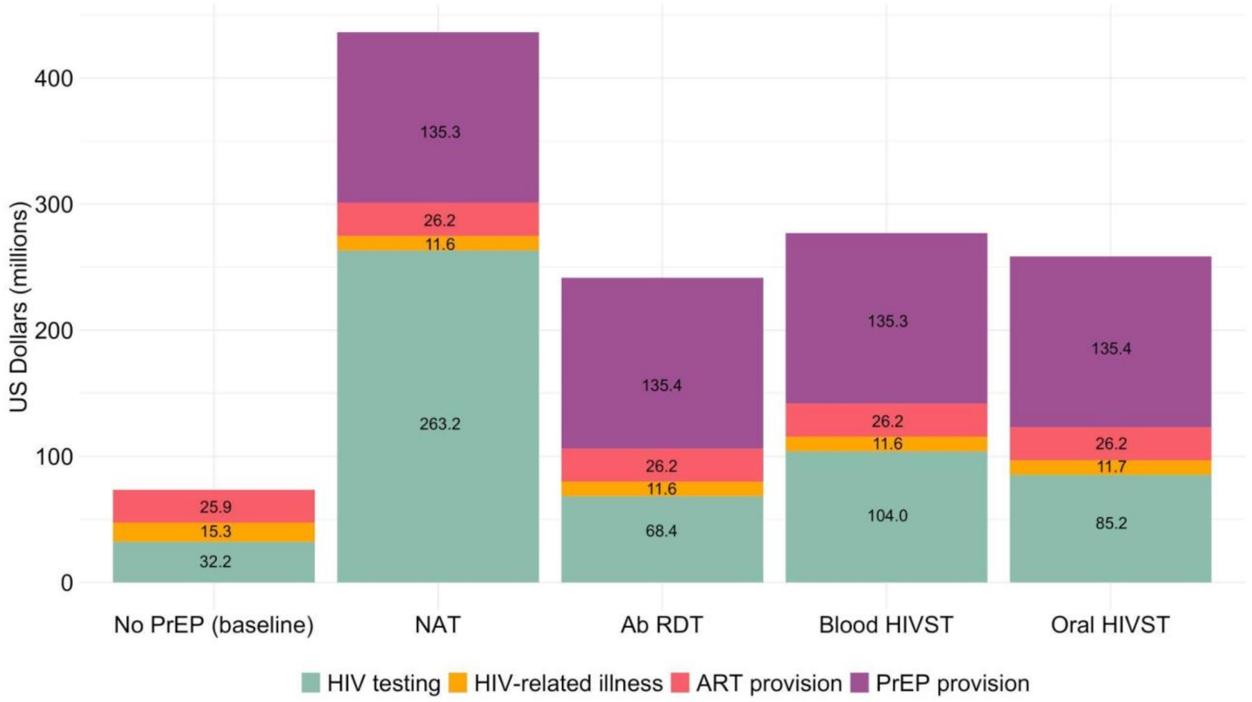

Figure S6b. Cost components of 5-year PrEP scale-up by HIV testing modality

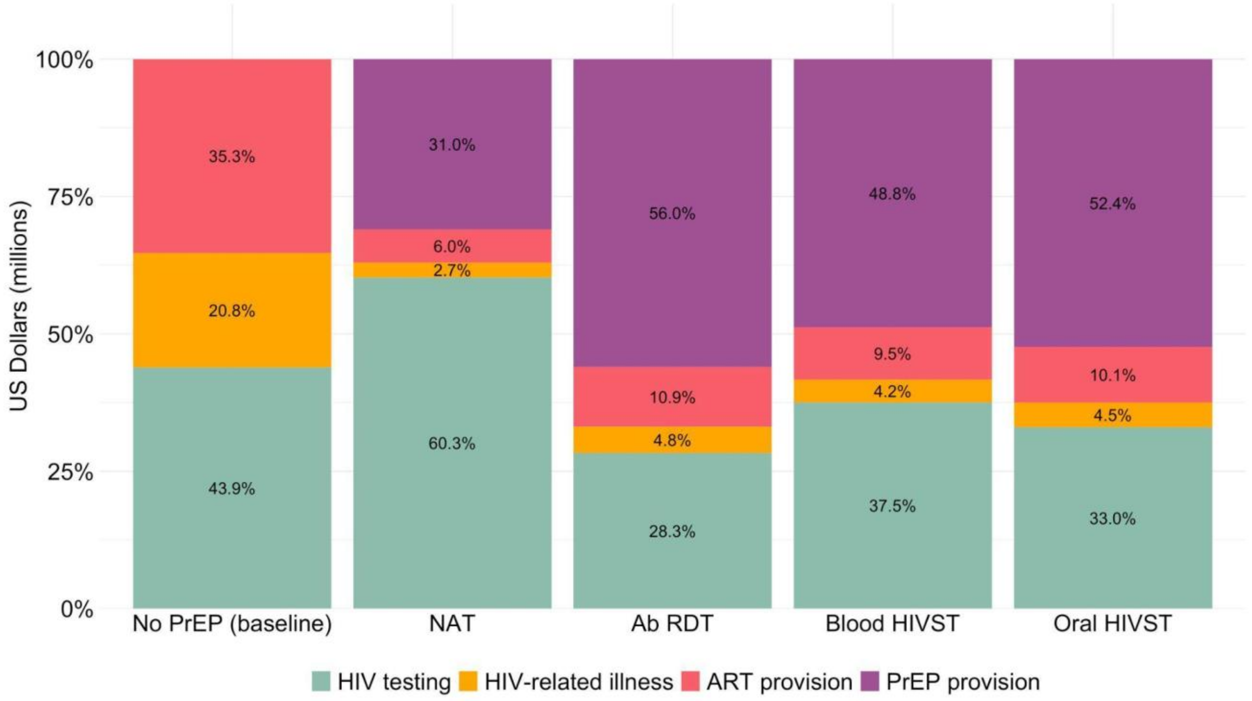

Figure S7. Modeled HIV incidence by age in western Kenya by scenario

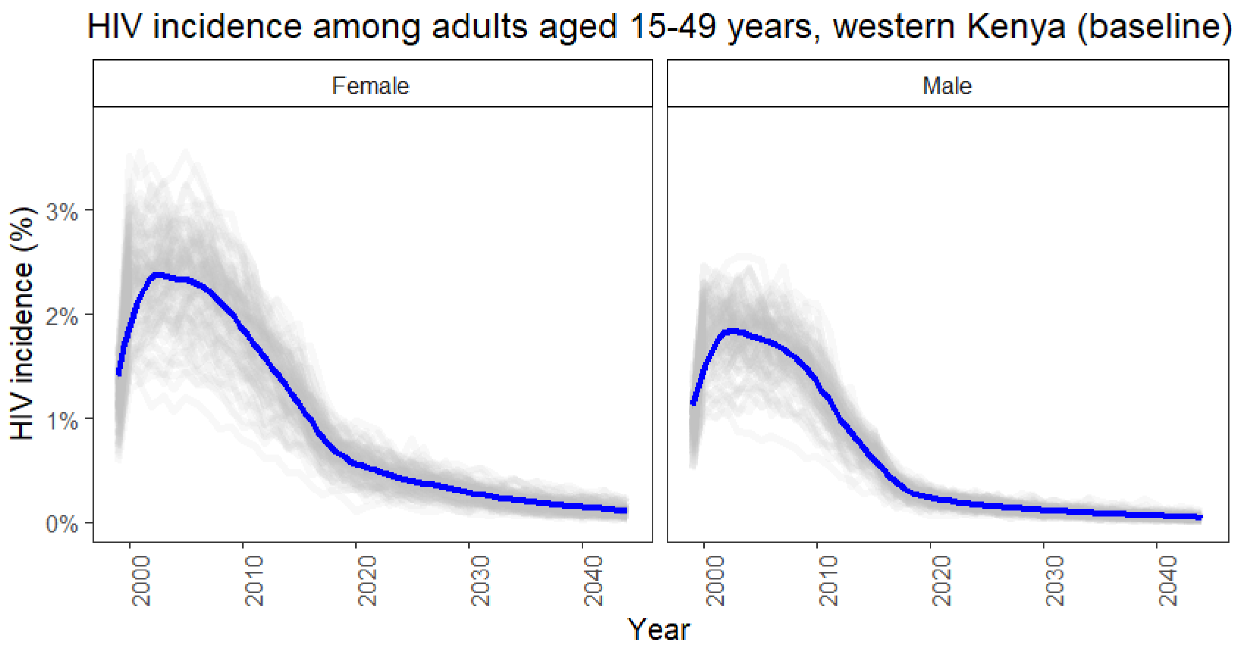

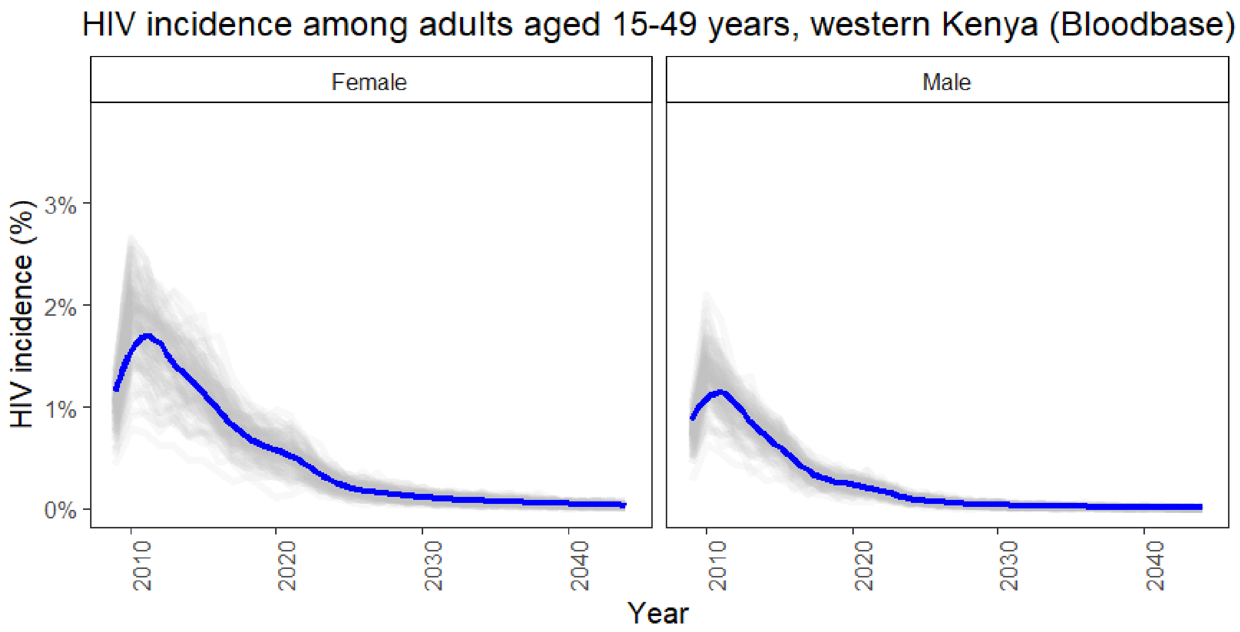

Figure S8. Modeled proportion of acute stage infections among persons not on ART in the population by scenario

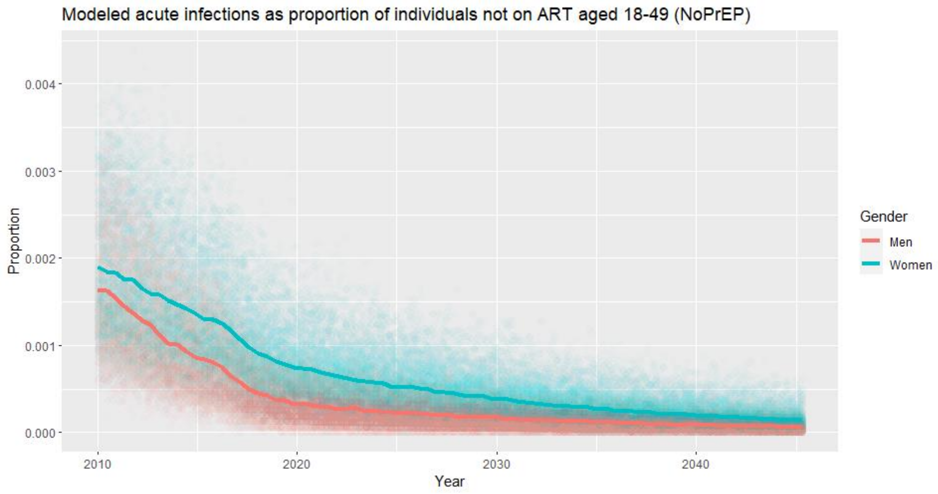

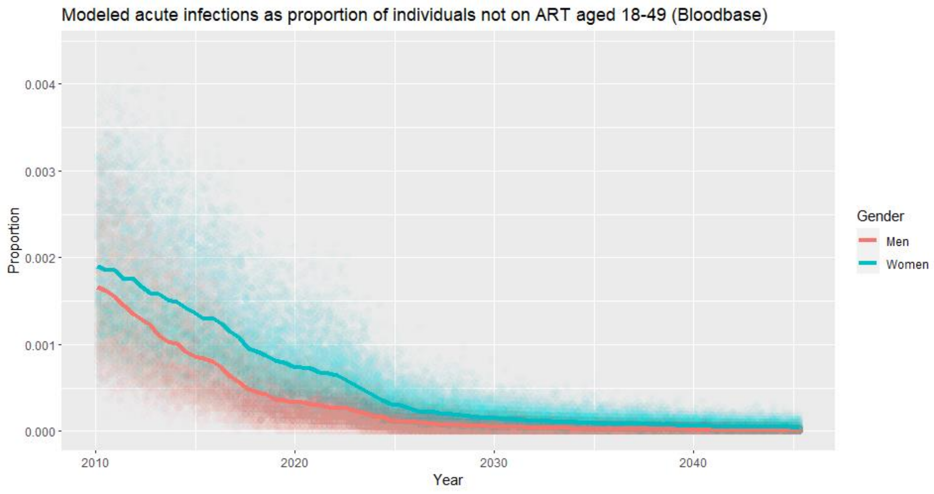

Figure S9. Modeled prevalence of population NRTI drug resistance by scenario

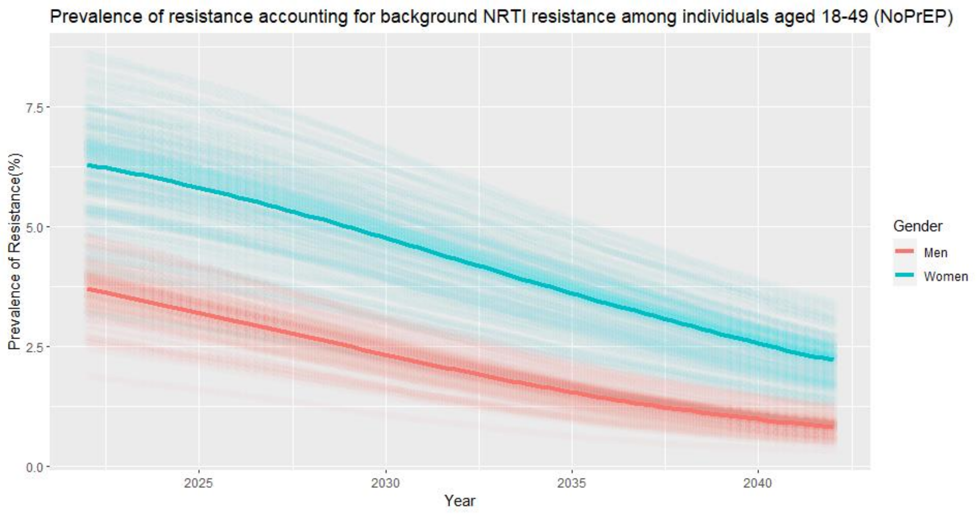

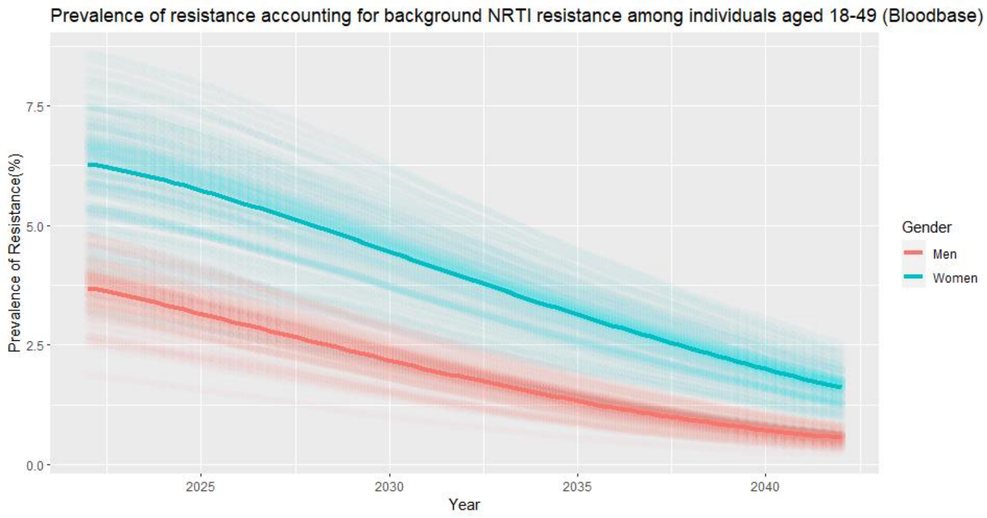

Probability of developing PrEP-related HIV drug resistance

To estimate the proportion of oral PrEP users developing PrEP-associated drug resistance, we conducted a scoping review of the clinical trial literature on oral PrEP efficacy relative to placebo or other HIV prophylaxis. We focus primarily on clinical trials since the level of data collected prospectively from baseline provides the most robust data on the etiologic process by which resistance develops. We included clinical trials of PrEP which tracked both patient HIV outcomes and resistance status from baseline throughout the trial duration. Studies were also included if they provided patient-level data, involved a TDF or TDF/FTC treatment arm, tested HIV status prospectively from baseline, and either prospectively or retrospectively assessed resistance status in patients determined to be HIV positive at any point during the follow-up period. We extracted and pooled individual data from each study regarding number of participants, number per trial arm, HIV status, and resistance status across time. Results of the literature review are summarized in Table S14.

We calculated prevalent (undiagnosed HIV at PrEP initiation) and incident (HIV negative at PrEP initiation) cases of HIV per arm and number of cases that subsequently developed an oral PrEP-associated mutation (M184I/V and/or K65R) (41,42,43) Results are calculated as a weighted average and presented in Table S15.

$$W = \sum_{i=1}^{n} w_i X_i$$

W represents a weighted average of the n=9 included studies, w_i is a study-specific weight calculated based on the number of individuals in the trial's TDF/FTC arm divided by the total number of individuals in TDF/FTC arm across all studies, and X_i is a study-specific estimate of proportion of oral PrEP initiators representing prevalent or incident cases, proportion of these cases estimated to subsequently develop a resistant mutation, or overall population-level estimate of the proportion of initiators expected to develop resistance.

Table S14. Clinical trials of oral PrEP report HIV infection, and resistance development pre- and post- randomization

Study	Setting	Year	Study Arm	N	Timing of HIV Infection		PrEP-selected resistance	
					Incident	Prevalent	Incident	Prevalent
Marzinke et al (50)	USA, S. America, Asia, Africa.	2021	TDF/FTC	2284	39	3	1	2
			CAB-LA	2282	12	4	12	2
Lehman et al (51)	Kenya, Uganda	2015	TDF	1584	30	8	1	1
			TDF/FTC	1579	21	4	1	2
			Placebo	1584	52	6	2	0
Grant et al (56)	South Africa, USA, Peru, Brazil, Thailand	2010	TDF/FTC	1251	36	8	0	2
			Placebo	1248	64	2	0	1
Thigpen et al (52)	Botswana	2012	TDF/FTC	611	9	1	0	1
			Placebo	608	24	2	0	1
Van Damme et al (57); Grant (58)	South Africa, Kenya, Tanzania	2012	TDF/FTC	1062	33	1	4	0
			Placebo	1058	35	1	1	0
Marrazzo et al (59)	South Africa, Zimbabwe, Uganda	2015	TDF	1007	52	5	0	0
			TDF/FTC	1003	61	9	1	2
			Placebo	1009	60	1	0	0
Choopanya et al (60)	Thailand	2013	TDF	1204	17	0	0	0
			Placebo	1209	33	2	0	0
Molina et al (61)	France, Canada	2015	TDF/FTC	199	2	1	0	0
			Placebo	201	14	1	0	0
McCormack et al (62)	United Kingdom	2016	TDF/FTC - Immediate	275	3	2	0	2
			TDF/FTC -Defer 1yr	269	20	1	0	0
Mayer et al (63)	US, UK, France, Germany, Spain	2020	TAF/FTC	2694	7	N/A	0	N/A
			TDF/FTC	2693	15	N/A	4	N/A

Evidence Synthesis

Of the nine included studies, there were 12,059 individuals in the TDF or TDF/FTC arms, of whom 43 (0.35%) were inappropriately on oral PrEP with an undetected HIV infection at initiation and 338 who acquired HIV post-PrEP initiation (2.5%). Of those inadvertently initiated on oral PrEP with an undetected HIV infection, 12 (33%) subsequently developed oral PrEP-associated resistance (Table S15). Of those acquiring HIV post-PrEP initiation, 12 (2.8%) developed oral PrEP resistance. Due to the high sensitivity of HIV tests utilized in the included clinical trials, no individuals with chronic HIV infection were initiated on PrEP. Therefore we assumed the probability of resistance development among these individuals would be half that of those with acute HIV infection, due to the lower viral load and selection pressure for resistance development (16.3%).

Table S15. Aggregated results indicating the proportion of PrEP initiators developing resistance

Proportion of oral PrEP initiators representing prevalent cases (undiagnosed acute HIV)	Of prevalent infections, proportion PrEP resistant (M184V/I or K65R)	Proportion of oral PrEP initiators representing incident cases (HIV negative at baseline)	Of incident infections, proportion PrEP resistant (M184V/I or K65R)
0.35% (n=43)	33.6% (n=12)	2.5% (n=338)	2.8% (n=12)

References

1. WHO. (2019). Consolidated guidelines on HIV testing services, 2019. Web Annex I. In vitro diagnostics for HIV diagnosis ISBN 978-92-4-001180-9. https://apps.who.int/iris/bitstream/handle/10665/335902/9789240011809-eng.pdf. Accessed 1 Sept. 2023.
2. Rosenberg, N. E., Pilcher, C. D., Busch, M. P., & Cohen, M. S. (2015). How can we better identify early HIV infections?. Current opinion in HIV and AIDS, 10(1), 61–68. https://doi.org/10.1097/COH.0000000000000121. Accessed 1 Sept. 2023.
3. Delaney, K., Violette, L., Ure II, G., Cornelius-Hudson, A., Niemann, L., Wesolowski, L., Chavez, P., Ethridge, S., McMahan, V., Clark, H., Katz, D., Stekler, J. (2018). Estimated time from HIV infection to earliest detection for 4 FDA-approved point-of-care tests.
4. WHO Prequalification of Diagnostics Programme. (2021). INSTI HIV Self Test. https://extranet.who.int/pqweb/sites/default/files/PQDx_0002-002-01_INSTI-HIV_SelfTest_v4.0.pdf. Accessed 1 Sept. 2023.
5. WHO Prequalification of Diagnostics Programme. (2021). OraQuick HIV Self-Test. https://extranet.who.int/pqweb/sites/default/files/PQDx_0159-055-01_OraQuickHIVSelfTest_v6.0.pdf. Accessed 1 Sept. 2023.
6. Pant Pai, N., Sharma, J., Shivkumar, S., Pillay, S., Vadnais, C., Joseph, L., Dheda, K., & Peeling, R. W. (2013). Supervised and unsupervised self-testing for HIV in high- and low-risk populations: a systematic review. *PLoS medicine*, *10*(4), e1001414. https://doi.org/10.1371/journal.pmed.1001414. Accessed 1 Sept. 2023.
7. Figueroa, C., Johnson, C., Ford, N., Sands, A., Dalal, S., Meurant, R., Prat, I., Hatzold, K., Urassa, W., & Baggaley, R. (2018). Reliability of HIV rapid diagnostic tests for self-testing compared with testing by health-care workers: a systematic review and meta-analysis. *The lancet. HIV*, *5*(6), e277–e290. https://doi.org/10.1016/S2352-3018(18)30044-4. Accessed 1 Sept. 2023.
8. Majam M, Fischer AE, Rhagnath N, Msolomba V, Venter WDF, Mazzola L, et al. Performance assessment of four HIV self-test devices in South Africa: A cross-sectional study. S Afr J Sci. 2021;117(1/2), Art. #7738. https://doi.org/10.17159/ sajs.2021/7738. Accessed 1 Sept. 2023.
9. Bwana, P., Ochieng', L., & Mwau, M. (2018). Performance and usability evaluation of the INSTI HIV self-test in Kenya for qualitative detection of antibodies to HIV. *PloS one*, *13*(9), e0202491.
10. Stekler, J. (2022). Expert Opinion
11. Neuman, M., Mwinga, A., Kapaku, K., Sigande, L., Gotsche, C., Taegtmeyer, M., Dacombe, R., Maluzi, K., Kosloff, B., Johnson, C., Hatzold, K., Corbett, E. L., & Ayles, H. (2022). Sensitivity and specificity of OraQuick® HIV self-test compared to a 4th generation laboratory reference standard algorithm in urban and rural Zambia. *BMC infectious diseases*, *22*(Suppl 1), 494. https://doi.org/10.1186/s12879-022-07457-5. Accessed 1 Sept. 2023.
12. Belete, W., Deressa, T., Feleke, A., Menna, T., Moshago, T., Abdella, S., Hebtesilassie, A., Getaneh, Y., Demissie, M., Zula, Y., Lemma, I., Mamo, G., Workalemahu, E., Kifle, T., & Abate, E. (2019). Evaluation of diagnostic performance of non-invasive HIV self-testing kit using oral fluid in Addis Ababa, Ethiopia: A facility-based cross-sectional study. *PloS one*, *14*(1), e0210866. https://doi.org/10.1371/journal.pone.0210866. Accessed 1 Sept. 2023.
13. Kurth, A. E., Cleland, C. M., Chhun, N., Sidle, J. E., Were, E., Naanyu, V., Emonyi, W., Macharia, S. M., Sang, E., & Siika, A. M. (2016). Accuracy and Acceptability of Oral Fluid HIV Self-Testing in a General Adult Population in Kenya. *AIDS and behavior*, *20*(4), 870–879. https://doi.org/10.1007/s10461-015-1213-9. Accessed 1 Sept. 2023.
14. Mavedzenge SN, Sibanda E, Mavengere Y, et al. Supervised HIV self-testing to inform implementation and scale up of self-testing in Zimbabwe. J Int AIDS Soc 2015; 18: 96.
15. Martínez Pérez, G., Steele, S. J., Govender, I., Arellano, G., Mkwamba, A., Hadebe, M., & van Cutsem, G. (2016). Supervised oral HIV self-testing is accurate in rural KwaZulu-Natal, South Africa. *Tropical medicine & international health : TM & IH*, *21*(6), 759–767. https://doi.org/10.1111/tmi.12703. Accessed 1 Sept. 2023.
16. Choko, A. T., MacPherson, P., Webb, E. L., Willey, B. A., Feasy, H., Sambakunsi, R., Mdolo, A., Makombe, S. D., Desmond, N., Hayes, R., Maheswaran, H., & Corbett, E. L. (2015). Uptake, Accuracy, Safety, and Linkage into Care over Two Years of Promoting Annual Self-Testing for HIV in Blantyre, Malawi: A Community-Based Prospective Study. *PLoS medicine*, *12*(9), e1001873. https://doi.org/10.1371/journal.pmed.1001873. Accessed 1 Sept. 2023.

17. Choko, A. T., Desmond, N., Webb, E. L., Chavula, K., Napierala-Mavedzenge, S., Gaydos, C. A., Makombe, S. D., Chunda, T., Squire, S. B., French, N., Mwapasa, V., & Corbett, E. L. (2011). The uptake and accuracy of oral kits for HIV self-testing in high HIV prevalence setting: a cross-sectional feasibility study in Blantyre, Malawi. *PLoS medicine*, *8*(10), e1001102. https://doi.org/10.1371/journal.pmed.1001102. Accessed 1 Sept. 2023.
18. Asiimwe, S., Oloya, J., Song, X., & Whalen, C. C. (2014). Accuracy of un-supervised versus provider-supervised self-administered HIV testing in Uganda: A randomized implementation trial. *AIDS and behavior*, *18*(12), 2477–2484. https://doi.org/10.1007/s10461-014-0765-4. Accessed 1 Sept. 2023.
19. Donnell, D., Ramos, E., Celum, C., Baeten, J., Dragavon, J., Tappero, J., Lingappa, J. R., Ronald, A., Fife, K., Coombs, R. W., & Partners PrEP Study Team (2017). The effect of oral preexposure prophylaxis on the progression of HIV-1 seroconversion. *AIDS (London, England)*, *31*(14), 2007–2016. https://doi.org/10.1097/QAD.0000000000001577. Accessed 1 Sept. 2023.
20. Hoornenborg, E., Prins, M., Achterbergh, R., Woittiez, L. R., Cornelissen, M., Jurriaans, S., Kootstra, N. A., Anderson, P. L., Reiss, P., de Vries, H., Prins, J. M., de Bree, G. J., & Amsterdam PrEP Project team in the HIV Transmission Elimination AMsterdam Consortium (H-TEAM) (2017). Acquisition of wild-type HIV-1 infection in a patient on pre-exposure prophylaxis with high intracellular concentrations of tenofovir diphosphate: a case report. *The lancet. HIV*, *4*(11), e522–e528. https://doi.org/10.1016/S2352-3018(17)30132-7. Accessed 1 Sept. 2023.
21. Landovitz, R. (2022, June 2). *Considerations on the use of RNA/VL as part of HIV Testing Algorithims with CAB-LA PrEP* [PowerPoint slides]. Center for Clinical AIDS Research and Education, UCLA.
22. Taylor, D., Durigon, M., Davis, H., Archibald, C., Konrad, B., Coombs, D., Gilbert, M., Cook, D., Krajden, M., Wong, T., & Ogilvie, G. (2015). Probability of a false-negative HIV antibody test result during the window period: a tool for pre- and post-test counselling. International journal of STD & AIDS, 26(4), 215–224. https://doi.org/10.1177/0956462414542987. Accessed 1 Sept. 2023.
23. Delaney, K. P., Hanson, D. L., Masciotra, S., Ethridge, S. F., Wesolowski, L., & Owen, S. M. (2017). Time Until Emergence of HIV Test Reactivity Following Infection With HIV-1: Implications for Interpreting Test Results and Retesting After Exposure. *Clinical infectious diseases: an official publication of the Infectious Diseases Society of America*, *64*(1), 53–59. https://doi.org/10.1093/cid/ciw666. Accessed 1 Sept. 2023.
24. PlotDigitizer. (2022). https://plotdigitizer.com. Accessed 20 February 2022.
25. D. J. Klein, "Relationship formation and flow control algorithms for generating age-structured networks in HIV modeling," 2012 IEEE 51st IEEE Conference on Decision and Control (CDC), Maui, HI, USA, 2012, pp. 1041-1046, doi: 10.1109/CDC.2012.6426573. Accessed 1 Sept. 2023.
26. Institutes for Disease Modeling. Relationships and contact networks — HIV Model documentation [Internet]. [cited 2023 Jul 23]. Available from: https://docs.idmod.org/projects/emod-hiv/en/latest/sti-model-relationships.html. Accessed 1 Sept. 2023.
27. Bershteyn A, Klein D. STI and HIV Model Introduction [Internet]. 2015 [cited 2023 Jul 23]. Report No.: IAS 2015. Available from: https://institutefordiseasemodeling.github.io/EMOD/STI_and_HIV_Tutorials.pdf. Accessed 1 Sept. 2023.
28. Ortblad, K. F., Bardon, A. R., Mogere, P., Kiptinness, C., Gakuo, S., Mbaire, S., Thomas, K. K., Mugo, N. R., Baeten, J. M., & Ngure, K. (2023). Effect of 6-Month HIV Preexposure Prophylaxis Dispensing With Interim Self-testing on Preexposure Prophylaxis Continuation at 12 Months: A Randomized Noninferiority Trial. JAMA network open, 6(6), e2318590. https://doi.org/10.1001/jamanetworkopen.2023.18590. Accessed 1 Sept. 2023.
29. Ortblad, K. F., Mogere, P., Roche, S., Kamolloh, K., Odoyo, J., Irungu, E., Mugo, N. R., Pintye, J., Baeten, J. M., Bukusi, E., Ngure, K., & Stakeholders for Pharmacy-based PrEP Delivery in Kenya Consultation (2020). Design of a care pathway for pharmacy-based PrEP delivery in Kenya: results from a collaborative stakeholder consultation. *BMC health services research*, *20*(1), 1034. https://doi.org/10.1186/s12913-020-05898-9. Accessed 1 Sept. 2023.
30. Roche, S. D., Wairimu, N., Mogere, P., Kamolloh, K., Odoyo, J., Kwena, Z. A., Bukusi, E. A., Ngure, K., Baeten, J. M., & Ortblad, K. F. (2021). Acceptability and Feasibility of Pharmacy-Based Delivery of Pre-Exposure Prophylaxis in Kenya: A Qualitative Study of Client and Provider Perspectives. AIDS and behavior, 25(12), 3871–3882. https://doi.org/10.1007/s10461-021-03229-5. Accessed 1 Sept. 2023.
31. Ekwunife O, Kuo A, Banerjee P, Kiptinness C, Omollo V, Chen Y, Roche S, Odoyo J, Bukusi E, Ngure K, Sharma M, Ortblad KF. Understanding the cost of pharmacy-delivered HIV pre-and post-exposure

prophylaxis service delivery in Kenya: findings from a pilot study. E-poster presentation at IAS 2023: 12th IAS Conference on HIV Science, Brisbane, Australia, July 23-26, 2023. Abstract EPE0887.

32. Eaton JW, Menzies NA, Stover J, Cambiano V, Chindelevitch L, Cori A, et al. Health benefits, costs, and cost-effectiveness of earlier eligibility for adult antiretroviral therapy and expanded treatment coverage: A combined analysis of 12 mathematical models. The Lancet Global Health [Internet]. 2014 Jan;2(1):e23–34. Available from: http://www.thelancet.com/article/S2214109X13701724/fulltext. Accessed 1 Sept. 2023.
33. Phillips AN, Cambiano V, Johnson L, Nakagawa F, Homan R, Meyer-Rath G, et al. Potential Impact and Cost-Effectiveness of Condomless-Sex-Concentrated PrEP in KwaZulu-Natal Accounting for Drug Resistance. The Journal of infectious diseases. 2021;223(8):1345–55.
34. Larson BA, Bii M, Henly-Thomas S, McCoy K, Sawe F, Shaffer D, et al. ART treatment costs and retention in care in Kenya: a cohort study in three rural outpatient clinics. Journal of the International AIDS Society [Internet]. 2013 Jan;16(1). Available from: /pmc/articles/PMC3536940/. Accessed 1 Sept. 2023.
35. The Global Fund. Pooled Procurement Mechanism Reference Pricing: ARVs [Internet]. [cited 2023 Jul 23]. Report No.: Version Q3 2023 – July 2023. Available from: https://www.theglobalfund.org/media/5813/ppm_arvreferencepricing_table_en.pdf. Accessed 1 Sept. 2023.
36. Kuo A, Ekwunife OI, Mogere P, Omollo V, Odoyo J, Chen Y, et al. Costs of providing pharmacy-initiated PrEP in Kenya: Findings from a pilot study [CROI Abstract 1090]. Abstracts From CROI 2023 Conference on Retroviruses and Opportunistic Infections CROI 2023 Abstract eBook 2023 [Internet]. Available from: https://www.croiconference.org/wp-content/uploads/sites/2/posters/2023/CROI2023_Costing_Poster_23.02.14-133209813359393813.pdf. Accessed 1 Sept. 2023.
37. Mangale D, Ortblad K, Heitner J, Mogere P, Kiptinness C, Mugo NR, et al. Comparing the cost of six-month PrEP dispensing with interim HIV self-testing to the standard-of-care three-month PrEP dispensing with clinic-based testing in Kenya. AIDS 2022 (Abstract # EPE237) [Internet]. Available from: https://programme.aids2022.org/PAGMaterial/PPT/1483_6781/Mangale_AIDS2022__e_poster_2022-07-17_FINAL.pdf. Accessed 1 Sept. 2023.
38. Meisner J, Roberts DA, Rodriguez P, Sharma M, Newman Owiredu M, Gomez B, et al. Optimizing HIV retesting during pregnancy and postpartum in four countries: a cost-effectiveness analysis. J Int AIDS Soc. 2021 Apr;24(4):e25686.
39. The Global Fund. Pooled Procurement Mechanism Reference Pricing: RDTs [Internet]. [cited 2023 Jul 23]. Report No.: Version: Q2 2023. Available from: https://www.theglobalfund.org/media/7564/psm_hivrdtreferencepricing_table_en.pdf. Accessed 1 Sept. 2023.
40. The World Bank. Inflation, consumer prices (annual %) - United States. [Internet]. [cited 2023 Jul 23]. files.https://data.worldbank.org/indicator/FP.CPI.TOTL.ZG?end=2021&locations=U. Accessed 1 Sept. 2023.
41. Gibas KM, van den Berg P, Powell VE, Krakower DS. Drug Resistance During HIV Pre-Exposure Prophylaxis. Drugs. 2019 Apr;79(6):609–19.
42. van de Vijver DAMC, Mukherjee S, van Kampen JJA. Antiretroviral Drug Treatment of Individuals that Used Preexposure Prophylaxis (PrEP) Before Diagnosis. Curr Treat Options Infect Dis. 2021 Sep 1;13(3):141–52.
43. Fonner VA, Dalglish SL, Kennedy CE, Baggaley R, O'Reilly KR, Koechlin FM, et al. Effectiveness and safety of oral HIV preexposure prophylaxis for all populations. AIDS. 2016 Jul 31;30(12):1973.
44. Parikh UM, Mellors JW. Should We Fear Resistance from Tenofovir/Emtricitabine PrEP? Curr Opin HIV AIDS. 2016 Jan;11(1):49–55.
45. Dimitrov DT, Boily MC, Hallett TB, Albert J, Boucher C, Mellors JW, et al. How Much Do We Know about Drug Resistance Due to PrEP Use? Analysis of Experts' Opinion and Its Influence on the Projected Public Health Impact. PLoS One. 2016 Jul 8;11(7):e0158620.
46. WHO recommends dolutegravir as preferred HIV treatment option in all populations [Internet]. [cited 2023 Feb 5]. Available from: https://www.who.int/news/item/22-07-2019-who-recommends-dolutegravir-as-preferred-hiv-treatment-option-in-all-populations

47. Ndashimye E, Arts EJ. Dolutegravir response in antiretroviral therapy naïve and experienced patients with M184V/I: Impact in low-and middle-income settings. Int J Infect Dis. 2021 Apr;105:298–303.

48. Phillips AN, Bershteyn A, Revill P, Bansi-Matharu L, Kripke K, Boily MC, et al. Cost-effectiveness of easy-access, risk-informed oral pre-exposure prophylaxis in HIV epidemics in sub-Saharan Africa: a modelling study. The Lancet HIV. 2022 May 1;9(5):e353–62.

49. Suntharasamai P, Martin M, Choopanya K, Vanichseni S, Sangkum U, Tararut P, et al. Assessment of Oral Fluid HIV Test Performance in an HIV Pre-Exposure Prophylaxis Trial in Bangkok, Thailand. PLoS One. 2015;10(12):e0145859.

50. Marzinke MA, Grinsztejn B, Fogel JM, Piwowar-Manning E, Li M, Weng L, et al. Characterization of Human Immunodeficiency Virus (HIV) Infection in Cisgender Men and Transgender Women Who Have Sex With Men Receiving Injectable Cabotegravir for HIV Prevention: HPTN 083. The Journal of Infectious Diseases. 2021 Nov 16;224(9):1581–92.

51. Lehman DA, Baeten JM, McCoy CO, Weis JF, Peterson D, Mbara G, et al. Risk of Drug Resistance Among Persons Acquiring HIV Within a Randomized Clinical Trial of Single- or Dual-Agent Preexposure Prophylaxis. The Journal of Infectious Diseases. 2015 Apr 15;211(8):1211–8.

52. Thigpen MC, Kebaabetswe PM, Paxton LA, Smith DK, Rose CE, Segolodi TM, et al. Antiretroviral Preexposure Prophylaxis for Heterosexual HIV Transmission in Botswana. New England Journal of Medicine. 2012 Aug 2;367(5):423–34

53. Pyra M, Brown ER, Haberer JE, Heffron R, Celum C, Bukusi EA, et al. Patterns of Oral PrEP Adherence And HIV Risk among Eastern African Women in HIV Serodiscordant Partnerships. AIDS Behav. 2018 Nov;22(11):3718–25.

54. MURNANE PM, CELUM C, MUGO N, CAMPBELL JD, DONNELL D, BUKUSI E, et al. Efficacy of pre-exposure prophylaxis for HIV-1 prevention among high risk heterosexuals: subgroup analyses from the Partners PrEP Study. AIDS. 2013 Aug 24;27(13):10.1097/QAD.0b013e3283629037.

55. Baeten JM, Donnell D, Ndase P, Mugo NR, Campbell JD, Wangisi J, et al. Antiretroviral Prophylaxis for HIV Prevention in Heterosexual Men and Women. New England Journal of Medicine. 2012 Aug 2;367(5):399–410.

56. Grant RM, Lama JR, Anderson PL, McMahan V, Liu AY, Vargas L, et al. Preexposure Chemoprophylaxis for HIV Prevention in Men Who Have Sex with Men. New England Journal of Medicine. 2010 Dec 30;363(27):2587–99.

57. Van Damme L, Corneli A, Ahmed K, Agot K, Lombaard J, Kapiga S, et al. Preexposure Prophylaxis for HIV Infection among African Women. New England Journal of Medicine. 2012 Aug 2;367(5):411–22.

58. Grant RM, Liegler T, Defechereux P, Kashuba ADM, Taylor D, Abdel-Mohsen M, et al. Drug resistance and plasma viral RNA level after ineffective use of oral pre-exposure prophylaxis in women. AIDS. 2015 Jan 28;29(3):331–7.

59. Marrazzo JM, Ramjee G, Richardson BA, Gomez K, Mgodi N, Nair G, et al. Tenofovir-Based Preexposure Prophylaxis for HIV Infection among African Women. New England Journal of Medicine. 2015 Feb 5;372(6):509–18.

60. Choopanya K, Martin M, Suntharasamai P, Sangkum U, Mock PA, Leethochawalit M, et al. Antiretroviral prophylaxis for HIV infection in injecting drug users in Bangkok, Thailand (the Bangkok Tenofovir Study): a randomised, double-blind, placebo-controlled phase 3 trial. Lancet. 2013 Jun 15;381(9883):2083–90.

61. Molina JM, Capitant C, Spire B, Pialoux G, Cotte L, Charreau I, et al. On-Demand Preexposure Prophylaxis in Men at High Risk for HIV-1 Infection. New England Journal of Medicine. 2015 Dec 3;373(23):2237–46.

62. McCormack S, Dunn DT, Desai M, Dolling DI, Gafos M, Gilson R, et al. Pre-exposure prophylaxis to prevent the acquisition of HIV-1 infection (PROUD): effectiveness results from the pilot phase of a pragmatic open-label randomised trial. The Lancet. 2016 Jan 2;387(10013):53–60.

63. Mayer KH, Molina JM, Thompson MA, Anderson PL, Mounzer KC, De Wet JJ, et al. Emtricitabine and tenofovir alafenamide vs emtricitabine and tenofovir disoproxil fumarate for HIV pre-exposure prophylaxis (DISCOVER): primary results from a randomised, double-blind, multicentre, active-controlled, phase 3, non-inferiority trial. Lancet. 2020 Jul 25;396(10246):239–54.
